# The solid and liquid states of chromatin

**DOI:** 10.1186/s13072-021-00424-5

**Published:** 2021-10-30

**Authors:** Jeffrey C. Hansen, Kazuhiro Maeshima, Michael J. Hendzel

**Affiliations:** 1 Department of Biochemistry and Molecular Biology, Colorado State University, Fort Collins, CO 80523 USA; 2 Genome Dynamics Laboratory, National Institute of Genetics, and Department of Genetics, Sokendai (Graduate University for Advanced Studies), Mishima, Shizuoka 411-8540 Japan; 3 Department of Cell Biology, Faculty of Medicine and Dentistry, University of Alberta, Edmonton, AB Canada; 4 Department of Oncology, Faculty of Medicine and Dentistry, University of Alberta, Edmonton, AB Canada

## Abstract

The review begins with a concise description of the principles of phase separation. This is followed by a comprehensive section on phase separation of chromatin, in which we recount the 60 years history of chromatin aggregation studies, discuss the evidence that chromatin aggregation intrinsically is a physiologically relevant liquid–solid phase separation (LSPS) process driven by chromatin self-interaction, and highlight the recent findings that under specific solution conditions chromatin can undergo liquid–liquid phase separation (LLPS) rather than LSPS. In the next section of the review, we discuss how certain chromatin-associated proteins undergo LLPS in vitro and in vivo. Some chromatin-binding proteins undergo LLPS in purified form in near-physiological ionic strength buffers while others will do so only in the presence of DNA, nucleosomes, or chromatin. The final section of the review evaluates the solid and liquid states of chromatin in the nucleus. While chromatin behaves as an immobile solid on the mesoscale, nucleosomes are mobile on the nanoscale. We discuss how this dual nature of chromatin, which fits well the concept of viscoelasticity, contributes to genome structure, emphasizing the dominant role of chromatin self-interaction.

## Background

Chromatin is the genetic material of eukaryotes. The core of a genomic chromatin fiber is an array of nucleosomes. Chromatin condenses the chromosomal DNA molecule into a globular territory in the nucleus. At the same time, chromatin is the substrate for functional processes such as transcription. This raises a fundamental question in chromatin biology. How is chromatin structured and packaged within a chromosome such that it can be accessed and navigated by proteins involved in DNA-based functions? Central to this question are the self-interacting properties of an array of nucleosomes, which dictate both the local packaging and global condensation of chromatin in vitro and in vivo. Any given stretch of genomic chromatin consists of a nucleosomal array bound to specific chromosomal proteins. Thus, in order to understand the structure and function of chromatin, one needs to understand both the fundamental behavior of an array of nucleosomes and how that behavior is influenced by the proteins and other factors that are bound to the nucleosomal array.

**Fig. 1:**
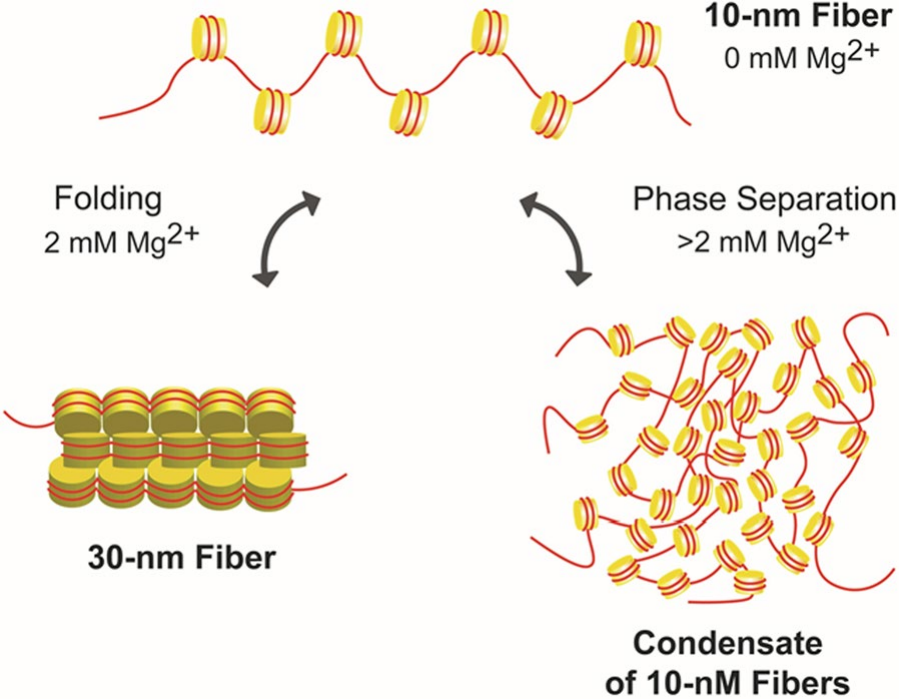
Schematic illustration of the different conformational states of an array of nucleosomes in solution. Depending on the extent of cation-dependent DNA charge neutralization, nucleosomal arrays can exist in an extended 10-nm conformation, a folded 30-nm conformation, or as a phase-separated condensate

The properties of chromatin in salt solutions have been actively investigated for 60 years. One aspect of the salt studies has been practical. Salts can be used to fractionate endogenous chromatin samples based on their propensity to aggregate. Another aspect of the salt studies has been analytical. Salts can be added back to chromatin samples to induce structural changes that subsequently are characterized by biochemical, biophysical, and microscopy methods. These studies have demonstrated that the chromatin fiber undergoes two structural transitions as salts are titrated into solution. One is a conformational change that involves local nucleosome–nucleosome interactions and results in formation of folded 30-nm fibers. The other is chromatin aggregation (Fig. 1). Beyond the salt range in which folding occurs, chromatin self-associates to form large aggregates that can be recovered as a pellet after centrifugation. This process is cooperative and reversible. Recent evidence suggests that chromatin aggregation is a phase separation phenomenon that can result in solid or liquid chromatin condensates depending on the solution conditions. The goal of this review is to critically discuss the phase separation behavior of chromatin and specific chromatin-associated proteins and relate this in vitro behavior to the properties of chromatin in the nucleus.

## Why do macromolecules phase separate?

Phase separation is a property of many biopolymers, including RNA, DNA, proteins, their complexes like chromatin. Phase separation is a thermodynamic process in which a mixture reaches its lowest free energy state by partitioning into a concentrated phase and a dilute phase [1–3]. The concentrated phase consists of aggregates of the solute(s), which are termed condensates. Condensates often have a globular or spherical morphology but can be irregular as well. There are both entropic and enthalpic contributions to a phase separation process. The entropy of mixing opposes phase separation; a well-mixed system is more disordered than a phase-separated system. Phase separation is promoted by the formation of non-covalent interactions between the solute molecules within the condensates. Biologically relevant non-covalent interactions include charge–charge, cation–pi, pi–pi, and dipole-based van der Waals interactions together with hydrogen bonds [4]. These attractive interactions make the enthalpy term negative and foster phase separation and condensate formation. If the solute–solute interactions are of high affinity, the condensates will have the material properties of a solid. If the interactions are weak and of low affinity, the condensates will behave as a liquid. Importantly, in the case of polyelectrolytes such as chromatin and RNA–protein complexes, the unfavorable enthalpy change resulting from charge repulsion between the polymer chains will oppose phase separation. Whether phase separation occurs under any given set of solution conditions depends on the overall sum of the favorable and unfavorable thermodynamic contributions. As with any association process, phase separation is sensitive to solute concentration. Dilute conditions favor the dispersed unassociated state, while phase separation and condensate formation occurs above a critical solute concentration [4]. Phase separation also is sensitive to the salt type and concentration. For proteins, lower levels of salt tend to promote phase separation, while higher levels disfavor phase separation by disrupting the electrostatic interactions (e.g., charge–charge, cation–pi) that stabilize the condensates. Chromatin and RNA–protein complexes differ in this respect. For theses macromolecules, cations are required to neutralize the negative charge of the nucleic acid, which reduces the magnitude of the charge repulsion term and thereby permits close packing of the complexes within the condensates. Interestingly, phase separation is often mediated by the intrinsically disordered regions of proteins, which tend to be capable of the complex polyvalent interactions found in condensates [5–8]. For example, the disordered N-terminal tail domains of the core histones are required for phase separation of chromatin, as will be discussed in detail below.

## Phase separation of chromatin

Salt-dependent aggregation of chromatin, which now is thought to be a phase separation process, has been studied since the 1950s, although for most of this time the phenomenon was considered to be irreversible precipitation of the chromatin from solution. We start this section by summarizing the historical development of chromatin aggregation research. We then highlight the data indicating that salt-dependent aggregation of chromatin fundamentally is a reversible LSPS process. This is followed by a survey of the molecular and macromolecular factors that influence chromatin LSPS, and a discussion of how the features of solid chromatin condensates mimic those of condensed chromatin in the nucleus. We conclude this section by highlighting the findings that salt-dependent chromatin aggregation produces liquid condensates under specific solution conditions.

### Sixty years of chromatin aggregation studies: from insoluble precipitants to phase-separated condensates

In 1957, Oth and Desreux [9] reported that endogenous preparations of chromatin are disperse in low ionic strength buffers but form high molecular weight aggregates in 0.15–0.4 M NaCl. At the time, chromatin was called nucleoprotein or nucleohistone and very little was known about it other than it contained DNA and equal amounts of the four core histones. A decade later, when describing the results of Oth and Desreux, Jensen and Chalkley [10] stated that the isolated chromosomal material “is precipitated from solution in the range of 0.15–0.40 M NaCl”. In their own studies, they defined “precipitated nucleohistone as that material sedimented …. from solution in 20 min at 23,500*g*.” Jensen and Chalkley [10] showed that endogenous preparations of sheared rat thymus chromatin could be separated into two different fractions based on their propensity to aggregate in 0.15 M NaCl. When discussing aggregation of nucleohistone in 0.15 M NaCl, Jensen and Chalkley stated that “the protein–DNA complex is negatively charged, and thus simple neutralization of charge might allow hydrophobic interactions between separate nucleohistone molecules to become more important than nucleohistone–water interactions. The result would be to aggregate molecules which would then fall out of solution.” [10]. These early studies helped established the concept that chromatin undergoes a salt-dependent transition from a soluble state to an insoluble precipitated state and that chromatin has different solubility under different salt conditions. This interpretation remains popular 60 years later.

Over the next 20 years, many studies exploited salt-dependent chromatin aggregation for the purposes of isolation and enrichment. Marushige and Bonner confirmed that after digestion with DNase II, rat liver chromatin could be separated into two fractions based on the aggregation of the released chromatin in 0.15 M NaCl [11]. The fraction of the chromatin sample that did not aggregate under these conditions was enriched in nonhistone proteins and RNA polymerases and had reduced amounts of the histones relative to DNA compared to the fraction that aggregated. As with the previous studies, the unaggregated chromatin fraction was described as “soluble” chromatin. Gottesfeld et al. examined the properties of the unaggregated and aggregated fractions of rat liver chromatin that were obtained after incubation in 2 mM MgCl_2_ [12]. The unaggregated fraction had the characteristics of transcriptionally active chromatin, while those of the aggregated fraction resembled bulk chromatin. These early studies were among the first to demonstrate that the physical properties of transcriptionally active chromatin are different than those of bulk chromatin, as manifested in the ability of the chromatin to aggregate in MgCl_2_. Davie and Candido [13] used the same fractionation protocol as Gottesfeld et al. [12] and found that the unassociated chromatin was enriched in acetylated histone H4, leading them to conclude that the H4 of active genes exists in a highly acetylated state. In their seminal paper, Perry and Chalkley examined the effects of histone hyperacetylation on chromatin aggregation and observed that hyperacetylated chromatin aggregated to a lesser extent in 5 mM MgCl_2_ than unacetylated chromatin, which was taken as reflecting increased solubility of the hyperacetylated chromatin [14]. Rocha et al. [15] used a fractionation scheme that involved sequential increases in NaCl [16] to show that nucleosomes released by MNase digestion from the active β-globin gene domain in chicken erythrocyte nuclei were selectively found in the unaggregated fractions at low salt, whereas the inactive ovalbumin and vitellogenin gene sequences were found predominantly in the aggregated chromatin fractions. Thirty years later, Henikoff et al. [17] performed genome-wide profiling on the chromatin fractions obtained by the method of Sanders [16]. Results indicated that the unaggregated fraction of nuclease-digested Drosophila chromatin obtained in 80 or 150 mM NaCl consisted of transcriptionally active sequences with unique chromatin signatures such as enrichment in H3.3 and H2A.Z. They concluded that salt fractionation provides a robust method for mapping genome dynamics. Salt-dependent chromatin aggregation continues to be utilized as a preparative method [18]. For example, Thakur and Henikoff combined salt fractionation with their CUT&RUN protocol to characterize the conformational variations of human centromeric chromatin [19]. Ultimately, while chromatin aggregation has been used as an effective fractionation tool in chromatin research for over 50 years, during this time there has been little interest in the physical nature of the aggregates themselves.

Analytical studies have characterized the structural changes that occur when NaCl or MgCl_2_ is added to chromatin preparations in very low salt [20, 21]. In 1979, Thoma et al. published their landmark paper on the salt-dependent structural changes of chromatin [22]. Although this paper is best known for its characterization of the folded 30-nm fibers that form when salts are first added to solution, and the role of linker histone H1 in the folding process, these investigators also noted that endogenous H1-containing chromatin forms aggregates at higher salt concentrations. Specifically, when describing the effects of NaCl on 30-nm fiber formation, they stated that, “No further change in morphology is observed on going to still higher ionic strengths (e.g., 100 mM NaCl + 50 mM sodium phosphate), but the solutions become turbid, indicating the onset of precipitation of the chromatin.” As discussed above, the interpretation that the chromatin aggregates are insoluble precipitants was commonplace at the time and often persists to this day. However, a turbid solution simply means the chromatin formed aggregates that were very large and readily scattered light. The chromatin fragments used by Thoma et al. [22] had an average length of 20–100 nucleosomes, equating to molecular masses of 5–25 MDa. In other words, the “monomers” in the chromatin aggregation reaction themselves were enormous. Consequently, it would be expected that the aggregates formed by such large chromatin fragments would turn the solutions turbid, regardless of their nature. The Thoma et al. paper [22] had a profound influence on the field. For the next 15 years, the focus of most analytical studies was on chromatin folding, while the chromatin aggregation process received much less attention.

**Fig. 2:**
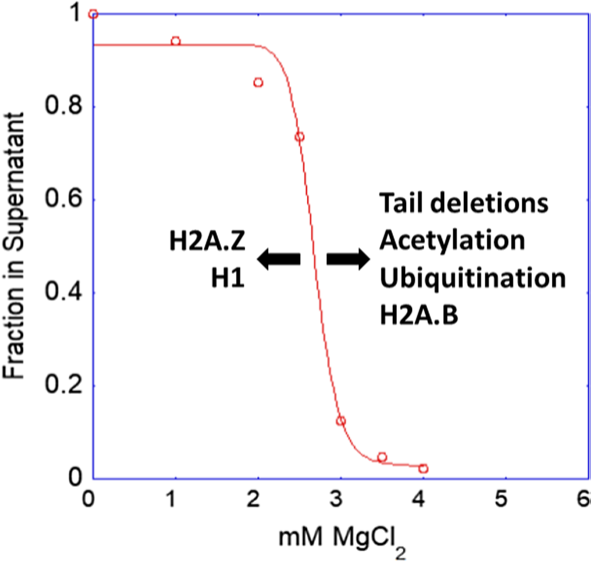
Information obtained from the differential centrifugation assay. In this assay, chromatin is combined with MgCl_2_ to the indicated value, pelleted in a microfuge for 10 min, and A_260_ of the supernatant measured. Data are expressed as the fraction of the chromatin in the supernatant as a function of MgCl_2_ concentration. Results are shown for native 12-mer nucleosomal arrays (red points and line). Factors that shift the native curve to the left or right are indicated

In 1985, Simpson and colleagues created DNA molecules consisting of tandem repeats of nucleosome positioning sequences, which could be reconstituted with purified core histones into nearly homogeneous preparations of defined nucleosomal arrays [23]. This innovation changed the face of the chromatin structure field and led to a much better understanding of the chromatin aggregation process. The effects of salts on the structure of the chromatin model systems were first examined in the late 1980s and early 1990s. In NaCl solutions, nucleosomal arrays formed moderately folded structures but did not aggregate [24, 25]. In contrast, in the presence of MgCl_2_, the nucleosomal arrays first folded into 30-nm fibers, then at higher salt concentrations formed aggregates [26]. The latter studies introduced the differential centrifugation assay as a tool for studying chromatin aggregation. In this assay, the chromatin sample is combined with salts and then centrifuged briefly in a microcentrifuge to pellet the aggregates. Data are expressed as the A_260_ of the supernatant as a function of salt concentration. This assay revealed that the aggregation of defined nucleosomal arrays with increasing MgCl_2_ concentration was a cooperative process (Fig. 2). Schwarz et al. [27] then reported two important findings. First, they showed that MgCl_2_-dependent aggregation of model nucleosomal arrays was reversible upon removal of the salt. Second, they observed that the core histone tail domains were required for nucleosomal arrays to aggregate—arrays reconstituted from trypsinized histone octamers lacking their tail domains did not aggregate, even at very high MgCl_2_ concentrations [27]. Tse et al. examined the effect of hyperacetylation of the core histone tails and found that acetylated nucleosomal arrays were still able to aggregate cooperatively, but at higher MgCl_2_ concentrations than control arrays [28] (Fig. 2). In contrast, binding of linker histone H1 to the nucleosomal arrays caused the arrays to aggregate in ≥ 100 mM NaCl and lowered the MgCl_2_ concentration at which aggregation was complete from 5 to 2 mM [29] (Fig. 2).

This initial series of model studies was entirely consistent with the chromatin aggregation literature and helped put the earlier results into perspective. For example, if endogenous chromatin produced by nuclease digestion was incubated in 150 mM NaCl, based on the model studies one would expect that most of the H1-containing chromatin fragments in the sample would form aggregates while the H1-depleted nucleosomal arrays in the sample would remain in the supernatant. In the studies of Tse et al. [28], control nucleosomal arrays were aggregated at MgCl_2_ concentrations where the hyperacetylated arrays remained completely unassociated, which is consistent with the observations that the unassociated chromatin fraction obtained in 150 mM NaCl or 2 mM MgCl_2_ is usually acetylated [14, 30, 31]. Importantly, despite the prevailing view that the aggregates were insoluble precipitants, the model studies showed that chromatin aggregation was a reversible, cooperative process that was mediated by the core histone tail domains and modulated by core histone acetylation and linker histones. As a consequence, Hansen [21] speculated that the nucleosomal arrays within the aggregated structures may interact with each other in the same way that the chromatin fiber interacts with itself over long distances in a condensed chromosome. Additionally, the aggregation process was called self-association or oligomerization rather than precipitation to better reflect that it is driven by reversible self-interaction of the chromatin fiber.

One of the most important papers in the chromatin aggregation literature was published in 2007 by Tremethick et al. [32]. These investigators reconstituted H2A.Z and H2A.B variant nucleosomal arrays and characterized their salt-dependent structural dynamics and ability to be transcribed by RNA polymerase II (Pol II) in vitro [32]. For the in vitro transcription experiments, nucleosomal arrays reconstituted from DNA templates containing the core HIV-1 promoter were first incubated in 6 mM MgCl_2_/72 mM NaCl, the conditions at which Pol II is maximally active. The arrays were then incubated with HeLa nuclear extract as a source of transcription factors and Pol II. All three types of arrays were completely aggregated in transcription buffer. Strikingly, all three types of arrays were transcribed by Pol II under these conditions, demonstrating that aggregated chromatin is a bona fide substrate for in vitro transcription. Their results also suggested that promoter sequences within the aggregated chromatin were accessible to exogenously added proteins. The results of Zhou et al. [32] further supported the interpretation that the packaging of the nucleosomal arrays within the chromatin aggregates is biologically relevant.

In retrospect, what was missing from all the early chromatin aggregation studies was knowledge of the physical properties of the aggregated structures. All that was really known was that the aggregates were very large, as reflected by the fact that they turn the solution turbid and pellet quickly in a microfuge. The dogmatic notion that chromatin has different solubility in different salts and that the aggregates represented an insoluble precipitated state of chromatin stunted physical studies of the aggregation process, despite the accumulated circumstantial evidence favoring its importance. During the last several years this situation has changed, and a clearer picture of the structure and properties of the chromatin aggregates has emerged. With it has come the realization that chromatin aggregation intrinsically is a reversible phase separation process that produces solid condensates with many of the features of condensed chromatin fibers in vivo. More recently, conditions have been found in which chromatin undergoes LLPS to generate liquid droplets [33]. Thus, condensed chromatin can exist in either a constrained solid-like state or a mobile liquid-like state in vitro depending on its environment.

### Chromatin aggregation intrinsically is a liquid/solid phase separation process

**Fig. 3:**
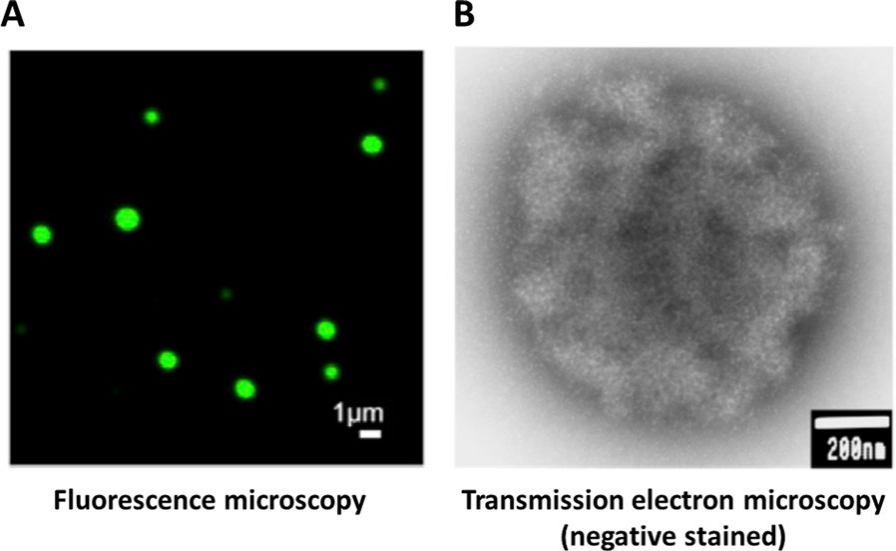
Visualization of chromatin condensates formed in MgCl_2_ by fluorescence and transmission electron microscopy. **A** Condensates were formed from Alexa 488-labeled 12-mer nucleosomal arrays in 4 mM MgCl_2_ and analyzed by fluorescence microscopy as described in reference [34]. Taken from Fig. 1D of reference [34] with permission. **B** Condensates were formed from 12-mer nucleosomal arrays in 5 mM MgCl_2_ and analyzed by transmission electron microscopy with negative staining as described in reference [34]

To better understand the physical features of chromatin aggregates, Maeshima et al. [34] used microscopy and physicochemical approaches to characterize the structures formed in MgCl_2_ by model 12-mer nucleosomal arrays with 60 bp linkers. These studies provided a number of fundamental insights into the chromatin self-association process. The first surprise was that the shape of the aggregates assembled from 12-mer arrays in 5 mM MgCl_2_ was not amorphous and irregular. Rather, the aggregates had a near spherical, globular morphology when observed by fluorescence microscopy (Fig. 3A). Transmission electron microscopy (TEM) also visualized globular structures (Fig. 3B). The negative staining used in the TEM experiments revealed that the surfaces of the chromatin globules were irregular and contoured rather than smooth. Both types of microscopy studies indicated that the chromatin globules had maximum diameters of 0.5–1.0 µm in 5 mM MgCl_2_, which is approaching the volume of a chromosome territory in the nucleus. Of note, native nuclease-digested chromatin fragments also form globular 0.5 µm diameter aggregates that further associate into complex 3D structures [35]. The very large size of the chromatin aggregates explains their ability to readily scatter light. Maeshima et al. [34] characterized the globular chromatin aggregates formed by 12-mer nucleosomal arrays using sedimentation velocity analytical ultracentrifugation and found that in 5 mM MgCl_2_ they sedimented as a heterogeneous population of particles with sedimentation coefficients ranging from 40,000–300,000S (by comparison, bacteriophage T7 sediments at 875S). Importantly, the Stokes radii of the largest globules calculated from the sedimentation coefficients were equivalent to the radii determined by microscopy, demonstrating that the globular chromatin assemblages observed by fluorescence and electron microscopy existed as stable entities in solution in the absence of cross-linking.

**Fig. 4:**
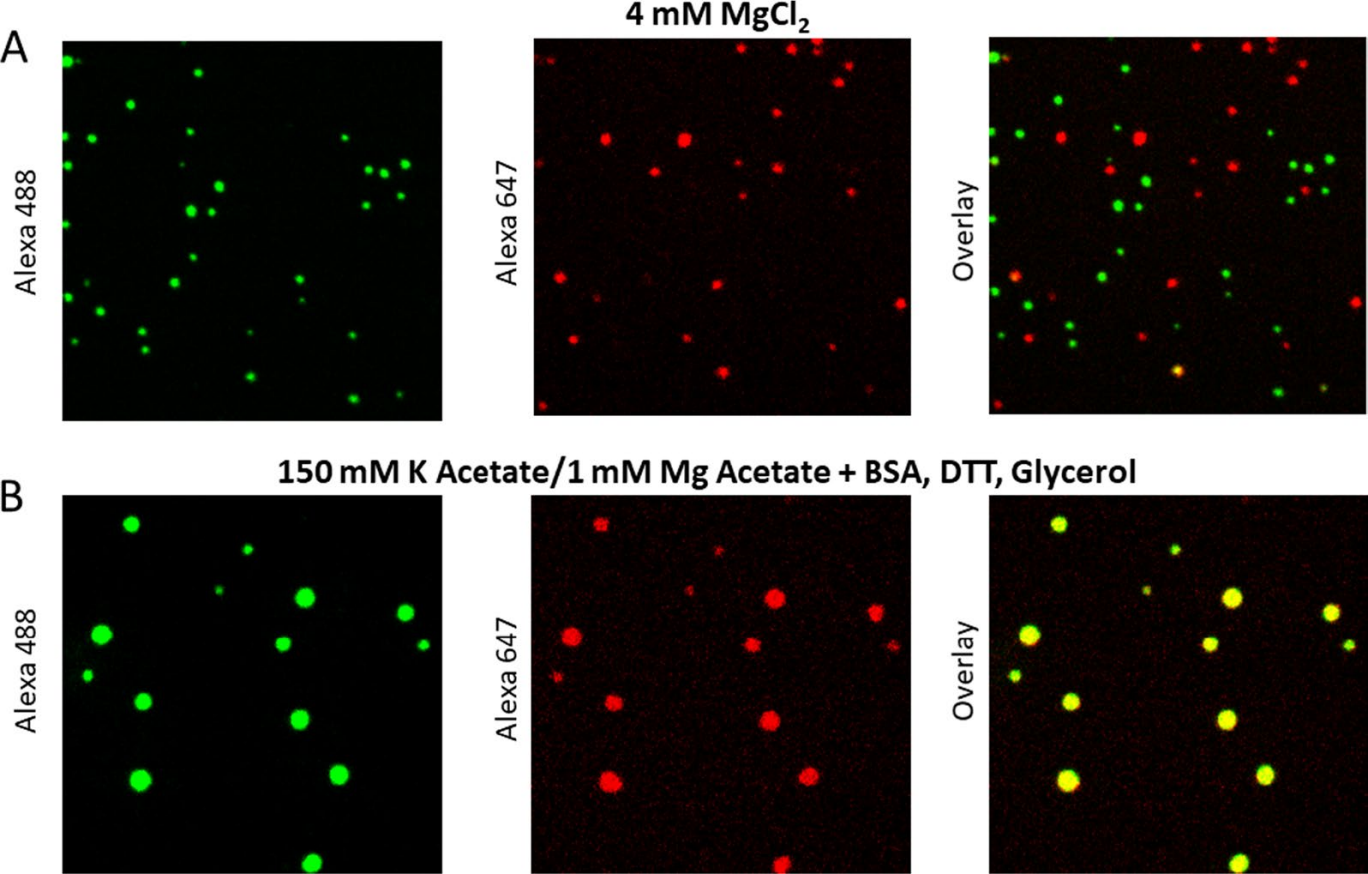
Two-color mixing assay for determination of the material state of chromatin condensates. 12-mer nucleosomal arrays (60 bp linkers) were reconstituted with recombinant Xenopus histone octamers in which histone H4 was labeled with Alexa 488 or Alexa 649. Labeled arrays were incubated in either 4 mM MgCl_2_ (**A**) or 150 mM K Acetate/1 mM Mg Acetate plus 0.1 mg/ml BSA, 5 mM DTT, and 5% glycerol (**B**) to form condensates. The green and red labeled condensates were then mixed for 20 min, followed by fluorescence microscopy. Shown are the images obtained in the green channel (left), red channel (center), and overlay (right) after the 20 min incubation. Data courtesy of Dr. Thomas Tolsma

The material state of the packaged nucleosomal arrays within the chromatin aggregates was determined by Strickfaden et al. [35], who tested whether the globules formed under various solution conditions were solid or liquid. Previous indirect observations were most consistent with a solid-like state. For example, in both the fluorescence microscopy and TEM images the aggregates appeared irregularly shaped [34]. Another characteristic of the aggregates is that they form discrete pellets upon low-speed centrifugation, which has been observed since the earliest studies of salt-dependent aggregation (see above). Strickfaden et al. [35] showed that the globules at high concentrations did not merge upon contact, instead forming 3D networks of interacting structures. They further found that globules formed from fluorescently labeled nucleosomal arrays in 4 mM MgCl_2_ failed to recover after they were photobleached in a FRAP experiment. Consistent with these findings, when red- and green-labeled condensates are formed in 4 mM MgCl_2_ and subsequently mixed, nucleosomal arrays do not exchange between the condensates (Fig. 4A). These observations indicate that the nucleosomal arrays within the chromatin condensates are packaged in a constrained, solid-like state with Mg^2+^. As will be discussed in the final section, there is evidence that condensed chromatin in vivo also has solid-like properties on the mesoscale [35].

The results of Maeshima et al. [34] and Strickfaden et al. [35] provided the evidence indicating that the phenomenon of reversible salt-dependent chromatin aggregation is a LSPS process, where the aggregates are the solid, globular condensates that make up the concentrated phase. As will be discussed below, the strong attractive intermolecular interactions that drive formation of the chromatin condensates are mediated by the disordered core histone N-terminal tail domains. Cations are required to achieve a critical level of DNA charge neutralization and decrease the electrostatic repulsion in the system, allowing the attractive interactions to dominate and shifting the overall balance toward phase separation. Importantly, chromatin aggregation is not a precipitation artifact caused by chromatin becoming insoluble at certain salt concentrations.

How are the nucleosomal arrays structured and packaged within the chromatin condensates? A nucleosomal array has an extended 10-nm fiber conformation in very low salt and folds into a 30-nm state in 1–2 mM MgCl_2_ ([26, 36]; see below). This raises the question of whether the nucleosomal arrays within the condensates are extended or folded. When the condensates formed in 5 mM MgCl_2_ were examined by TEM at high magnification a mass of closely packed nucleosomes could be seen, but no regular structures were visualized [34]. Small angle X-ray scattering (SAXS) can detect repetitive structures within complex biological macromolecules [37], including chromatin and chromosomes [38]. In control experiments performed with dispersed nucleosomal arrays in 1.0 and 2.5 mM MgCl_2_, a 30-nm peak was present in the SAXS data [34]. In contrast, when the condensates formed in 5 mM MgCl_2_ were examined, peaks were observed at 6 and 11 nm, but not at 30 nm [34]. Thus, both the TEM images and the SAXS data showed that the nucleosomal arrays within the chromatin condensates did not have a regular helical 30-nm structure. A 6-nm peak in the SAXS curve results from face-to-face nucleosome–nucleosome packing while the 11-nm peak reflects edge-to-edge nucleosome–nucleosome packing [38], indicating that both types of nucleosome arrangements are present within the chromatin condensates. The simplest explanation for the TEM images and SAXS profiles is that the nucleosomal arrays are packaged within the condensates as irregular 10-nm zig-zag fibers that interdigitate with one another [34]. Importantly, considerable evidence has accumulated suggesting that chromatin in bulk is packaged within chromosomes in the same manner as the nucleosomal arrays within the condensates (see below).

### Factors that control chromatin LSPS

One essential determinant of chromatin LSPS that has already been mentioned is salt. Other factors are intrinsic to the chromatin itself, such as the core histone tail domains, the nucleosome acidic patch, and linker DNA. Most of what is known about the determinants of chromatin LSPS has come from older studies employing the differential centrifugation assay. Thus, while many factors have been identified that influence the onset of chromatin LSPS, in most cases we do not know how these factors affect the structural features of the condensates.

#### Salts and the polyelectrolyte effect

Nucleosomal arrays aggregate in 5 mM MgCl_2_ (ionic strength, *I*, equals 15 mM) and 5 mM MgCl_2_/100 mM NaCl (*I* = 115 mM), but not in 115 mM NaCl (*I* = 115 mM). This behavior is inconsistent with chromatin becoming insoluble at certain ionic strengths. Instead, the effects of salt on LSPS can be explained by the polyelectrolyte properties of chromatin [39]. A polyelectrolyte is a polymer whose repeating monomer unit is charged. DNA is a polyelectrolyte with high negative charge density (2 negative charges/0.34 nm). Consequently, DNA in aqueous solution attracts cations, which neutralize a significant fraction of its negative charge. Divalent and multivalent cations physically bind to DNA and are most effective at neutralizing DNA charge. Monovalent cations are loosely associated with DNA and are less effective at charge neutralization. When ~ 90% of the negative charges are neutralized by cations, DNA forms condensates through a phase separation process [39]. This critical level of charge neutralization is achieved by multivalent cations (e.g., polyamines, oligolysine) but not by monovalent or divalent cations [39]. Chromatin also is a polyelectrolyte by nature of its DNA component. As with naked DNA, LSPS of chromatin occurs when ~ 90% of the DNA charge in the system is neutralized [39]. Screening of DNA charge by cations decreases the magnitude of the unfavorable enthalpic term resulting from charge repulsion, tipping the equilibrium toward phase separation and condensate formation. From a practical standpoint, this extent of charge neutralization permits close packing of the chromatin within the condensates. Because the highly basic histones themselves neutralize a large fraction (~ 55%) of DNA negative charge [39], LSPS of nucleosomal arrays can be induced by physiological concentrations of divalent cations, and monovalent cations if linker histones are bound to the arrays (see below). In addition, the histones—in the form of regularly spaced nucleosomes—create geometrical constraints that give the chromatin condensates important biological characteristics, e.g., interdigitated nucleosome packaging.

#### Core histone N-terminal tail domains

Each of the core histones has a highly positively charged and disordered N-terminal “tail” domain that projects away from the nucleosome. The tail domains are required for LSPS of chromatin. Early studies showed that nucleosomal arrays assembled from trypsinized histone octamers lacking their tail domains failed to undergo LSPS, even at 50 mM MgCl_2_ [27, 28]. Subsequent investigations used the differential centrifugation assay to examine all 15 different combinations of recombinant tailless nucleosomal arrays [40]. All nucleosomal arrays lacking one or two sets of tail domains formed pelletable condensates, but at higher MgCl_2_ concentrations than the wild-type control (Fig. 2). The finding that increased salt could replace the missing tails indicates that the tails promote LSPS in part by binding to DNA and neutralizing its negative charge. This is logical as the tails are complex polyvalent cations. Detailed studies of H4 tail mutants confirmed the dominant role of DNA charge neutralization in chromatin LSPS [41]. Gordon et al. [40] further showed that nucleosomal arrays containing only the H3 or H4 tails could form pelletable condensates, but arrays containing only the H2A or H2B tails could not. These results indicate that some property of the H3 and H4 tails is required for LSPS that is not shared by H2A and H2B and that cannot be replaced by salt. We speculate that this property is the formation of the inter-array cross-links that stabilize the chromatin condensates. Finally, Gordon et al. [40] demonstrated that the core histone tails act additively and independently of one another when mediating chromatin LSPS, consistent with the tails functioning as autonomous DNA binding modules. The conclusions of Gordon et al. [40] were supported by the work of Hayes and colleagues, who used chemical cross-linking to probe the contacts made by the H3 and H4 tail domains within the chromatin condensates [42–45]. Intriguingly, these studies demonstrated that only ~ 20% of the total H3 and H4 tail cross-links with DNA were inter-array [42]. Thus, only a subset of the H3 and H4 tails are engaged in inter-array interactions within the condensates. We speculate that strong [46] inter-array interactions involving the H3 and H4 tails are responsible for the solid-like features of the chromatin condensates on the mesoscale and cannot be replaced with salt. The remaining 80% of the H3 and H4 tail–DNA cross-links were bound to the linker DNA of their own arrays [42–44], where they neutralize DNA charge as discussed above.

#### Post-translational modifications

Consistent with the essential functions of the tail domains in mediating chromatin LSPS, condensate formation can be regulated by tail post-translational modifications. The best studied modification is acetylation. Acetylation adds two carbons to the lysine side chain—capping it with a methyl group—while abolishing a positive charge. Acetylation is often described as increasing the solubility of chromatin [14]. However, acetylation makes chromatin more hydrophobic and less charged, both of which will *decrease* its solubility in aqueous solution. The early work of Perry and Chalkley demonstrated that native hyperacetylated chromatin stays in the supernatant at MgCl_2_ concentrations at which unacetylated chromatin pellets in the centrifuge [14, 30]. Tse et al. [28] confirmed and extended these observations by showing that acetylated nucleosomal arrays were able to form condensates, but at higher MgCl_2_ concentrations than wild-type nucleosomal arrays (Fig. 2). Moreover, the amount of extra MgCl_2_ needed to induce LSPS was directly proportional to the extent of acetylation and reduction in tail positive charge [28]. The same result was obtained in a detailed analysis of specific H4 tail acetylations by Allhverdi et al. [47], who also showed that lysine → glutamine mutations gave the same results as acetylations. Dhall et al. [48] studied the effects of H4K12ac and H4K16ac on chromatin LSPS and found that they were equivalent. Abolishing tail positive charges lessens the degree of DNA charge neutralization by the tails. Together, these studies indicate that acetylation shifts the onset of LSPS to higher MgCl_2_ concentrations by modulating the polyelectrolyte properties of chromatin and increasing the amount of charge repulsion in the system, not by increasing chromatin solubility. Shogren-Knaak et al. [49] reported that acetylation of lysine 16 in the H4 tail (H4K16ac) had the same effect on LSPS as complete removal of the H4 tail, which at first glance is at odds with the other studies. However, this result can be explained if H4K16ac led to complete dissociation of the H4 tail from DNA under the conditions employed by Shogren-Knaak [49]. Mishra et al. [50] probed the effect of H3 and H4 tail acetylations on the local packaging of nucleosomal arrays within chromatin condensates. In this work, nucleosomal arrays were constructed in which a single nucleosome bearing lysine → glutamine acetylation mimics in the H4 tail was inserted into the middle of a 25-mer array, followed by condensate assembly in 10 mM MgCl_2_ and restriction enzyme digestion to probe linker DNA accessibility. When the four lysines in the H4 tail were replaced with glutamine, the linker DNA surrounding the mutated tail became more accessible to restriction digestion within the assembled condensates. Thus, a single nucleosome with “hyperacetylated” H4 tails is capable of locally disrupting the interdigitated packaging of the nucleosomal arrays in a sea of condensed chromatin.

Modification of H4K12 by the addition of ubiquitin or the ubiquitin-like protein SUMO increased the MgCl_2_ concentration at which LSPS occurs [48] (Fig. 2), consistent with neutralization of the H4K12 charge by the modifications. What is interesting in this case is that the nucleosomal arrays are able to form condensates even though small globular proteins are covalently attached to the H4 tails. Fierz et al. [51] examined condensate formation by nucleosomal arrays containing ubiquitin linked to H2BK120, a residue present on the nucleosome surface that is ubiquitylated in vivo. Ubiquitylation shifted the onset of LSPS to higher MgCl_2_ concentrations to the same extent as H4K16ac. However, the effects of H2BK120ub and H4K16ac were additive, suggesting that these two modifications influence condensate assembly through different mechanisms. When ubiquitin was replaced with a similar protein Hub1, the onset of LSPS was not affected, ruling out a steric effect on array packaging. Rather, Debelouchina et al. [52] showed that the mechanism involved ubiquitin–ubiquitin interactions mediated by two acidic residues on the protein surface.

All told, other than the effects of acetylation and to some extent ubiquitylation, very little is known about how histone post-translational modifications influence chromatin LSPS, and almost nothing is known about how modifications affect the structure and packaging of the nucleosomal arrays within the condensates. Future studies addressing these questions will contribute significantly to our understanding of the structural basis of open and closed chromatin.

#### Nucleosome acidic patch

The surface of the nucleosome has a cluster of aspartate and glutamate residues from H2A and H2B that are collectively known as the acidic patch [53]. When chromatin is in the folded 30-nm state, the H4 tails of a given nucleosome are bound to the acidic patch of its nucleosome neighbors [54]. H4 tail-acidic patch interactions also occur within the chromatin condensates, although they are not required for LSPS to occur, and their functional role remains to be clarified. If the charge patch is disrupted by replacing acidic residues with neutral residues, *less* MgCl_2_ is required to induce LSPS [32, 55, 56] (Fig. 2). Interestingly, the H4 tail-acidic patch interaction is modulated naturally by the histone variants H2A.Z and H2A.B. The acidic patch formed by the H2A.Z variant has fewer negatively charged residues than wild type, while that formed by H2A.B is expanded. H2A.Z and H2A.B arrays undergo LSPS at lower and higher MgCl_2_ concentrations than wild type, respectively [32, 55]. These results suggest that inhibition of the H4 tail-acidic patch interaction frees up more H4 tails to bind to DNA, leading to more DNA charge neutralization and less MgCl_2_ needed for LSPS. Conversely, when the acidic patch is expanded, fewer H4 tails are bound to DNA and more MgCl_2_ is required for LSPS. Sinha and Shogren-Knaak [57] demonstrated that under MgCl_2_ conditions that induced LSPS, at least some of the H4 tail-acidic patch contacts within the condensates were between arrays. Kan et al. [42] also observed that a fraction of the H4 tails cross-link to the H2A of other arrays. Taken together, it appears that the acidic patch does not directly participate in chromatin LSPS, but indirectly influences the process by acting as a sink for the H4 and possibly other tails.

#### Linker histones

Chromatin in the nucleus consists of a host of proteins bound to the underlying nucleosomal array. The most common chromatin protein in higher eukaryotes is histone H1 [58]. Nucleosomal arrays bound to H1 undergo LSPS in 150 mM NaCl and at lower MgCl_2_ concentration than nucleosomal arrays alone as determined by the pelleting assay [29, 59] (Fig. 2), although H1-bound nucleosomal arrays do not form condensates if the core histone tails are missing [60]. The effect of H1 is mediated largely by its carboxyl-terminal domain (CTD) with some contribution from the globular domain and/or amino- terminal domain (NTD) [59]. The CTD is disordered, very basic, and binds to linker DNA, further highlighting the important role of DNA charge neutralization in chromatin LSPS. The H1-bound arrays are packaged within the condensates as 10-nm fibers [34]. SAXS data suggest that H1 reduces the local mobility of the arrays within the condensates and that the H1-bound arrays are extensively interdigitated with each other [34]. Consistent with conclusion, Mishra and Hayes [61] demonstrated that linker DNA is much less accessible within the condensates formed by H1-bound arrays compared to those formed by control nucleosomal arrays. Of interest, the condensates formed by H1-bound arrays are smaller than those formed by nucleosomal arrays alone at equivalent MgCl_2_ concentrations [34], although the meaning of this result is unclear because the factors that control condensate size are not understood at this time. Taken together, the effects of linker histones on chromatin LSPS indicate that H1 stabilizes the interdigitated packaging of nucleosomal arrays within chromatin condensates, leading to decreased linker DNA accessibility and possibly formation of more densely packed structures.

### Are the chromatin condensates formed by LSPS physiologically relevant?

The single long chromatin fiber that makes up a chromosome in the nucleus does not exist in a dispersed state, but rather is extensively condensed due to self-interaction over long distances. Formation of chromatin condensates also is driven by chromatin self-interaction. This raises the question whether the structural features of the chromatin within the condensates mirror the structural features of condensed chromatin in the nucleus. Condensed euchromatin and heterochromatin domains behave as solids on the mesoscale in vivo, as do chromatin condensates in vitro [35]. Condensed chromatin in vivo exists in bulk as irregular 10-nm zig-zag fibers or clusters of nucleosomes [62]. Likewise, the nucleosomal arrays within chromatin condensates adopt a 10-nm conformation, both in the absence and presence of histone H1[34]. SAXS studies of intact nuclei yield the same 6-nm and 11-nm peaks as observed for chromatin condensates, and the SAXS profile of the condensates formed by H1-bound nucleosomal arrays is virtually identical to the SAXS profile of isolated HeLa nuclei, suggesting that the 10-nm chromatin zig-zags are interdigitated in condensates in vitro and in interphase chromosomes in vivo. The linker DNA within the chromatin condensates is accessible to exogenously added micrococcal nuclease [34] and restriction enzymes [50, 61], like condensed chromatin in vivo. Chromatin condensates can be transcribed by RNA polymerase II in vitro [28, 32], indicating that the linker DNA is also accessible to the HeLa nuclear extract proteins needed for transcription, many of which are very large macromolecular complexes. Moreover, these studies indicate that the chromatin condensates can support a key functional process that takes place in a condensed chromatin environment in vivo. As discussed in the previous sections, histone acetylation, H2A variants, and histone H1 all affect the formation of chromatin condensates in vitro and are associated with regulation of chromatin condensation in vivo. Chromatin condensate formation is sensitive to the Mg^2+^ concentration in vitro. Increased Mg^2+^ concentrations in the nucleus resulting from ATP hydrolysis leads to increased chromatin condensation in vivo [63]. If chromatin condensates are formed in MgCl_2_ and then returned to low-salt buffer, the condensates become unstable and disassemble into non-interacting 10-nm fibers. Incubation of isolated HeLa nuclei in low-salt buffer leads to massive chromatin decondensation and complete disruption of nuclear ultrastructure in situ [34]. By all of these criteria, the properties of chromatin condensates formed with Mg^2+^ in vitro closely mimic the properties of bulk condensed chromatin in the nucleus.

### Liquid/liquid phase separation of chromatin

Under specific solution conditions, cations will induce LLPS rather than LSPS. This phenomenon was first reported by Gibson et al. [33] and also observed by Strickfaden et al. [35]. Rather than using MgCl_2_ to induce phase separation, Gibson et al. [33] incubated 12-mer nucleosomal arrays in buffers containing 1 mM Mg acetate/150 mM K acetate plus several additives, including glycerol, dithiothreitol (DTT) and bovine serum albumin (BSA). Under these conditions, the nucleosomal arrays formed condensates that were large, spherical, and merged upon contact. In FRAP experiments, rapid fluorescence recovery was observed after bleaching of an internal portion of the condensate, indicative of movement of the nucleosomal arrays within the condensates. If the mixing assay shown in Fig. 4 is performed under the conditions of Gibson et al. [33], the nucleosomal arrays within the red and green condensates freely exchange during the 20-min incubation, forming large yellow condensates (Fig. 4B). These properties indicate that the chromatin condensates are liquid droplets rather than solid globules under specific solution conditions. Strickfaden et al. [35] subsequently showed that the formation of liquid chromatin droplets required the combination of BSA, DTT, and acetate anions. Removal of any one of these components from the buffer yielded condensates that were solid as judged by FRAP. The native conformation of BSA is stabilized by many disulfide bonds, suggesting that some property of reduced BSA is required to convert cation-driven chromatin LSPS to a LLPS process. Upon reduction with DTT, native BSA is transformed into a molten globule-like state characterized by increased surface hydrophobicity [64]. Strickfaden et al. [35] proposed that the targets of the additives are the strong tail–DNA interactions that stabilize solid chromatin condensates. We speculate that reduced BSA and acetate create conditions in which the tail–DNA interactions are weak and transient rather than strong and stable, leading to a packaged chromatin state that is liquid. It should be noted that the liquid chromatin droplets have high internal viscosity and are not particularly fluid [33]. Of note, it has recently been demonstrated that the material state of DNA-based condensates is sensitive to the DNA fragment length [65]. For both H1–DNA condensates and nucleosomal arrays, shorter (< 1 kb) DNAs formed liquid condensates while longer fragments formed more solid condensates.

For 12-mer nucleosomal arrays, the chromatin condensates formed by LSPS and LLPS behave similarly in some respects and differently in others. Removal of the core histone tails, and disruption of tail DNA-contacts, abolishes formation of both solid and liquid condensates [33]. As discussed above, the tail–DNA interactions are likely to be low affinity and fluctuating in liquid condensates and more stable in the solid state. The solid chromatin condensates characterized by Strickfaden et al. [35] had 60 bp linkers. Systematic decreases in linker DNA length have little effect on chromatin LSPS; nucleosomal arrays with linkers ranging from 20–60 bp all formed pelletable condensates at nearly the same MgCl_2_ concentration [66]. Some of these arrays had 10n bp linkers and some had 10n + 5 bp linkers. In contrast, Gibson et al. [33] found that nucleosomal arrays with 10n bp linkers required a higher salt concentration to form liquid condensates compared to 10n + 5 bp linker arrays [33]. The 10n + 5 condensates had higher fluorescence intensity as well, together indicating that nucleosomal arrays with 10n linkers interact differently within liquid condensates than 10n + 5 linkers. The results of Gibson et al. [33] indicate that linker DNA length will be an effector of liquid chromatin properties assuming that conditions exist that promote LLPS of chromatin in vivo.

Gibson et al. [33] devised an inducible system to examine the effect of acetylation on chromatin LLPS. In their experiments they attached the catalytic domain of p300 to an *E. coli* transcription factor and then inserted the transcription factor binding site into the middle of their nucleosomal arrays. This allowed for p300-dependent acetylation of the nucleosomal arrays upon incubation with acetyl CoA. Liquid chromatin condensates were first formed from unmodified nucleosomal arrays, followed by addition of acetyl CoA. Over the next 25 min the condensates dissolved and eventually disappeared, demonstrating that acetylation is capable of the disrupting chromatin LLPS [33]. It was not determined whether acetylation failed to cause droplet dissolution at higher Mg^2+^ concentrations, as would be predicted from the LSPS results.

Linker histone-bound nucleosomal arrays form solid condensates in MgCl_2_ that are smaller than those formed by nucleosomal arrays alone [34]. The same phenomenon occurs with the liquid chromatin condensates formed by LLPS [33]. In the case of the solid condensates, linker histones lead to changes in SAXS profiles that are consistent with reduced local mobility of the nucleosomal arrays [34]. Interestingly, under conditions that produce liquid condensates of nucleosomal arrays, linker histone-bound nucleosomal arrays form solid condensates as judged by lack of recovery in a FRAP experiment [33]. Thus, linker histones strengthen nucleosomal array–nucleosomal array interactions within both liquid and solid chromatin condensates [34, 61]. For both LSPS and LLPS, the effects of linker histones are mediated by the long intrinsically disordered H1 CTD [33, 60].

As will be discussed in the final section, the condensed chromatin found in in heterochromatin and euchromatin domains in the nuclei of living cells is solid on the mesoscale [35]. As such, the properties of the solid chromatin condensates formed by LSPS are most relevant to understanding the properties of bulk condensed chromatin in the nucleus (see above). At the same time, the results of Gibson et al. [33] and Strickfaden et al. [35] raise the question whether conditions exist in vivo that mimic the conditions that support chromatin LLPS in vitro. If this is the case, any given specific region of chromatin in the nucleus may exist in a liquid state.

## Phase separation of chromatin-binding proteins

One paradox of nuclear organization is that macromolecules can enrich within subregions of the interphase nucleus despite the absence of membrane barriers to diffusion [67]. This is particularly confounding for nuclear compartments that exclude or contain very little chromatin, such as the PML body, splicing factor compartment, and nucleolus. Phase separation provides a plausible mechanism to establish and maintain this organization and has become a very active area of investigation. LLPS of nuclear proteins garnered initial interest with the demonstration that poly(ADP-ribose) assembled at sites of laser-induced DNA damage initiated liquid–liquid unmixing to form a phase-separated liquid compartment responsible for the retention of specific proteins at the DNA damage site [6]. Evidence for poly(ADP-ribose) stimulated phase separation built on an expanding body of literature surrounding proteins with prion-like domains and mutations in RNA binding proteins that lead to neurodegenerative disease where LLPS is a physiological state and the “hardening” or gelling of these structures into solid-like structures is pathophysiological [68, 69].

**Fig. 5:**
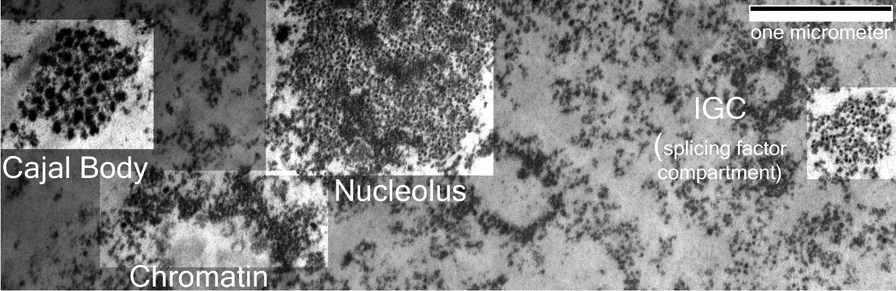
Nuclear bodies visualized by transmission electron microscopy. A K562 cell nucleus obtained by fixation with 4% paraformaldehyde and embedding in Epon 812 was imaged by electron spectroscopic imaging at 250 eV energy loss. This generates high contrast images of the biological specimen where contrast is related to the mass density. The image shows examples of condensed chromatin, the nucleolus, a Cajal body, and an interchromatin granule cluster (splicing factor compartment). The brightness was increased in the regions containing these structures to highlight their locations. The classification is based upon their morphologies in the transmission electron microscope. The scale bar represents 1 µm

Since the initial observation of PARP-dependent phase separation at DNA damage sites, phase separation has been used to explain the formation and maintenance of PML bodies, Cajal bodies, nucleoli and splicing factor compartments [8, 70]. Consistent with the hypothesis that nuclear bodies represent condensed phases of specific nuclear proteins and nucleic acids, reportedly phase-separated nuclear bodies typically have a much higher apparent molecular density in TEM images (Fig. 5). Molecules within these compartments are expected to experience increased molecular crowding. Macromolecular crowding can influence the structure of both folded and intrinsically disordered proteins, favoring a more compact conformation, but the influence on specific proteins can vary [8]. Conformational changes induced by crowding could have functional significance providing regulatory mechanisms beyond controlling local reactant concentrations. In a crowded environment, diffusion-limited reactions are expected to occur at reduced rates due to reduced diffusion in the condensate, while those limited by transition kinetics are stimulated [71].

Two types of LLPS phenomena have been observed that could reflect an important role for LLPS in regulating chromatin organization and function in living cells. In the first case, liquid–liquid unmixing is an intrinsic property of specific chromatin or chromatin-associated proteins that phase separate above a critical concentration when present in solutions of defined ionic strength and pH. This property has been observed for purified histones [72, 73] and members of the Chromobox (CBX) family of heterochromatin-associated proteins [74–78]. In the second case, LLPS is only observed when the chromatin-binding protein is mixed with DNA, nucleosomes, or nucleosomal arrays. An example of this type of behavior is MeCP2 [79–81]. Both types of condensates can incorporate nucleosomal arrays, which is consistent with the potential for liquid condensates enriched in heterochromatin proteins to recruit and concentrate chromatin [74, 76]. Thus, recent models propose that the formation of LLPS condensates comprised heterochromatin-binding proteins promotes the compaction of chromatin [82, 83].

### Histones

The core histones contain intrinsically disordered amino-terminal domains that contribute to the assembly of higher-order chromatin structures (discussed above). Histone H1 has an unstructured, low complexity, highly basic C-terminal domain that constitutes approximately half the total mass of H1 [84]. The H1 C-terminal domain undergoes LLPS when mixed with a 20 bp oligonucleotide [72, 73]. Increasing the salt concentration reversed LLPS, and phosphorylation of the H1 CTD inhibited LLPS [72, 73]. Interestingly, H1 also forms condensates with single stranded DNA and RNA [85]. Droplet formation as a function of histone concentration was studied in 150 mM NaCl. Histones H1 and H2B, but not histones H2A, H3, or H4, formed condensates when mixed with DNA that could be reversed by high salt concentrations that dissociate histone–DNA complexes (400 mM NaCl) [72, 73]. The other core histones formed irreversible precipitates when mixed with DNA under these conditions. No molecular crowding agents were used in these studies. When H1 and H2A were added to nucleosomal arrays in 150 mM NaCl, the mixtures assembled into irregularly shaped condensates, consistent with formation of a solid-like state [73, 86]. As discussed above, nucleosomal arrays alone are dispersed in 150 mM NaCl while H1-bound nucleosomal arrays undergo LSPS at this salt concentration due to the increased DNA charge neutralization afforded by the H1 [29]. Both Maeshima et al. [33] and Gibson et al. [35] showed that the condensates formed by H1-bound nucleosomal arrays behave as a solid. Thus, the results obtained in 150 mM NaCl by Shakya et al. [73] are consistent with these earlier studies. However, mechanistically H1 functions by neutralizing DNA charge and promoting the intrinsic phase separation of nucleosomal arrays, not by forming proteinaceous condensates that incorporate nucleosomal substrates.

### Heterochromatin-binding proteins

There are two major heterochromatin classes, constitutive heterochromatin marked by H3K9 trimethylation and facultative heterochromatin marked by H3K27 trimethylation. Both types of heterochromatin form visibly condensed structures within interphase nuclei as judged by TEM [87, 88]. If chromatin-binding proteins are capable of undergoing LLPS in isolation and then bind to specific histone modifications they might coalesce the modified chromatin into a liquid condensate, and they could initiate the formation of the dense chromatin structure that makes up heterochromatin. Several genetically and biochemically well-characterized heterochromatin proteins have been studied using the liquid droplet assay for LLPS. CBX5/HP1α (constitutive heterochromatin) and CBX2 (facultative heterochromatin) both undergo LLPS in buffers containing near-physiological concentrations of monovalent ions [74–77]. These heterochromatin proteins recognize either trimethylated histone H3K9 [89, 90] or trimethylated histone H3K27 [91] through their chromodomain, respectively.

HP1 proteins can undergo both LLPS independent of chromatin and can incorporate chromatin into pre-existing condensates. For example, purified HP1a, the Drosophila homolog, forms liquid droplets at approximately 10 μM in 50 mM NaCl and 20  μM at 100 mM NaCl [75]. Similarly, CBX5/HP1α was shown to form condensates in buffers containing 75 mM KCl, 20 mM HEPES pH 7.2, and 1 mM DTT [74]. The ability of CBX5/HP1α to form condensates was dependent upon phosphorylation and required DNA binding sequences only found in the CBX5 variant of HP1. Moreover, the addition of DNA stimulated condensate formation without the requirement for phosphorylation, and CBX5/HP1α condensates could recruit nucleosomes containing histone H3 trimethylated on lysine 9 [74]. However, phase separation of CBX5/HP1α occurs at concentrations of at least 45 μM [92]. Larson and colleagues argue that binding to closely spaced nucleosomes will increase local concentrations to approximately 100 μM [74]. Erdel and colleagues estimate the concentration of CBX5/HP1α to range between 1 and 3 μM within constitutive heterochromatin [92]. Thus, there is considerable uncertainty about whether HP1 reaches a sufficient concentration to undergo LLPS [92], particularly in regions where it has not been concentrated by chromatin.

A related family of HP1-related chromobox proteins recognizes lysine 27 methylation [91]. Polycomb group proteins mediate H3K27 methylation and are recognized by polycomb group chromodomain-containing CBX proteins The chromodomains within CBX2, 4, 6,7, and 8 recognize the methylation imparted by the EZH2 methyltransferase subunit of polycomb repressive complex 1 [91]. The CBX subunit is part of the polycomb repressive complex 1, which deposits ubiquitin on lysine 119 of H2A as part of the polycomb transcriptional repression process [93]. Polycomb group proteins localize to discrete nuclear bodies, termed polycomb group bodies [94]. Two groups recently demonstrated that CBX2 undergoes liquid–liquid phase separation in vitro and proposed LLPS as a mechanism for forming polycomb group bodies [76, 77]. In isolation, CBX2 forms droplets at concentrations as low as 2.5 μM in buffers containing 100–150 mM K/NaCl with or without 1 mM MgSO_4_ at pH 7.4–7.9 [76, 77]. When CBX2 was mixed with other members of the polycomb repressive complex (Ring1a, PCH2, BMI1), the entire PRC1 complex also formed liquid droplets [76]. This lower critical concentration for LLPS suggests the potential for condensate formation independent of chromatin, whereas this seems less likely with CBX5/HP1α.

Because CBX proteins can undergo LLPS in vitro, this raises the question: are these condensates compatible with chromatin? HP1 condensates have been studied in mixtures with chromatin. It is important to note that, in these experiments, the influence of buffer conditions on the state of the chromatin itself is not considered. There are subtle differences in condensate behavior among the different chromatin mixing studies, which highlights the potential impact of the environmental conditions on the properties of these condensates. In one study, H3K9me3 peptides, H3K9me3-containing nucleosomes, and H3K9me3-containing nucleosomal arrays all formed condensates when mixed with CBX5/HP1α [74]. In a subsequent study, the interactions of the *S. pombe* homologue, Swi6, with nucleosomes and nucleosomal arrays were characterized [95]. Mixtures of Swi6 (2 µM) and nucleosomal arrays (40 nM) formed liquid droplets under ionic conditions where the arrays themselves remain dispersed in solution (150 mM KCl, 10 mM Tris–Cl pH 7.8, 0.1 mM EDTA). Notably, while lysine 9 trimethylation reduced the concentration of Swi6 necessary to form liquid condensates by approximately half, Swi6 promoted condensate formation with unmodified arrays as well [95]. However, a second study demonstrated that HP1α only formed condensates efficiently in the presence of H3K9me3-containing arrays and not wild-type arrays. In this instance, the array concentration (33 µM) was similar to the HP1α concentration (12.5 to 50 µM) and condensates were observed at concentrations as low as 12.5 µM [96]. Although the two studies employed similar buffer conditions, the stoichiometry of the nucleosomal arrays and HP1 was markedly different. H3K9me3 specificity was observed when the ratio of Swi6 to nucleosomal arrays was much closer to unity (12.5 µM Swi6, 8 µM histone H3). Interestingly, the Arabidopsis homologue of HP1 (ADCP1) alone does not form condensates. Instead, ADCP1 only forms condensates in the presence of nucleosomal arrays [78].

There is also evidence that the liquid protein condensates formed by polycomb group proteins can incorporate chromatin. Plys et al. [76] found that arrays containing H3K27me3 reduced the concentration of CBX2 necessary to form condensates. Tatavosian et al. [77] reported that CBX2 condensates could concentrate nucleosomes, but mutations of the chromodomain to prevent interaction with H3K27me3 did not prevent assembly into polycomb group bodies within cells. Moreover, loss of H3K37me3 in *Eed* null cells did not prevent the assembly of CBX2 into polycomb group bodies in cells. This indicates that in the much more intricate environment found in the cell, and while functioning as part of a protein complex (PRC1), additional interactions may be responsible for CBX2 retention in polycomb group bodies. For example, the polymerization of the sterile alpha motif (SAM domain) within Polyhomeotic was shown to be required for clustering of PRC1 subunits in cells [97]. Polyhomeotic is part of the canonical polycomb repressive complex 1, conserved from flies to humans, consisting of a chromobox (CBX) subunit, that has specificity for histone H3K27me3, a PCGF (PCGF1-6)/RNF (Ring1a, RNF2) E3 ubiquitin ligase and a polyhomeotic subunit (HPH) [98]. PRC1 is responsible for the ubiquitylation of histone H2A at lysine 119, commonly enriched in facultative heterochromatin [93, 99, 100]. The SAM domain of Polyhomeotic was recently shown to support phase separation in the presence of DNA or reconstituted nucleosomal arrays [101]. In this instance, the nucleosomal arrays were reconstituted onto a circular plasmid containing 40 Lytechinus 5S rDNA nucleosome positioning sequences. Condensates formed at concentrations as low as 630 nM of the SAM-domain and 40 nM of the nucleosomal array. Notably, condensate formation stimulated H2A ubiquitylation in vitro, and overexpression of the SAM domain stimulated H2A ubiquitylation in cells [101]. Interestingly, when array mobility within the condensates was assessed in 50 mM NaCl Tris–Cl pH 8.0, the tagged SAM domain was found to move relatively freely, whereas the labeled nucleosomal arrays recovered very little. Moreover, experiments revealed that labeled and unlabeled chromatin condensates mixed poorly [101]. Thus, the chromatin condensates formed in the presence of the SAM domain behaved as if they were more solid than liquid.

In contrast to CBX proteins, MeCP2, a 5MeC DNA binding protein that also associates with heterochromatin, undergoes LLPS but *only* in the presence of DNA or nucleosomal arrays. MeCP2 was tested for liquid droplet formation in buffers containing 100–150 mN NaCl. Liquid droplets did not form unless DNA was added [79–81]. These studies also incorporated micromolar concentrations of MeCP2 with DNA concentrations being at least tenfold lower. Nucleosomes and nucleosomal arrays were also sufficient to induce phase separation with MeCP2 when MeCP2 was in molar excess [79–81, 102]. Interestingly, in the presence of methylated DNA, a 4X nucleosome array forms condensates at concentrations of MeCP2 as low as 160 nM when the array is present at 112.5 nM [81]. This is approaching physiological concentrations of nucleosomes [103, 104]. MeCP2 mutations are associated with Rett syndrome, a neurodevelopmental disease that leads to severe neurological impairment. All three groups found that mutations found in patients in the DNA binding domain or the intrinsically disordered region containing the transcriptional repression domain reduced the ability of MeCP2 to form condensates in vitro and reduced partitioning to heterochromatin in cells. However, it should be cautioned that these same mutations influence many other aspects of MeCP2 function including the ability to recruit co-repressors.

Mixtures of chromatin and chromatin-binding proteins potentially represent a better environment to study the physiological role of LLPS. One recent study looked at how a network of phase-separated proteins contributes to phase separation [96]. Both H3K9 methyltransferase SUV39H1 and HP1 have chromodomains that bind to H3K9me3. They showed that a tetrameric complex of two SUV39H1 and two HP1β (CBX1) proteins could concentrate H3K9me3 chromatin and form condensates when mixed with nuclear extracts containing large chromatin fragments. They determined that at least three chromodomains were required in the complex to form condensates efficiently. They further identified separate interactions between HP1β and SUV39H1 and HP1β and TRIM28 and demonstrated they acted cooperatively to induce phase separation in mixtures with reconstituted nucleosomal arrays containing H3K9me3, but not wild-type nucleosomal arrays [96]. These experiments illustrate how combinations of proteins, acting cooperatively, may lower the concentration thresholds determined for individual proteins to form condensates, at least in vitro.

These assays have significant limitations that need to be considered when extrapolating their results to potential roles in organizing or compacting chromatin to the heterochromatin compartments observed in vitro. Relative to the interphase nucleus, these experiments typically involve much higher protein to nucleosome ratios, lower to much lower chromatin concentration, and much less conducive conditions for LSPS of chromatin. Thus, a more physiological in vitro assay would incorporate buffer conditions that promote chromatin LSPS, lower concentrations of chromatin-binding proteins, and higher chromatin concentrations. For example, while these studies generally have been performed in near-physiological levels of monovalent cations, they commonly lack the divalent cations that help drive LSPS of chromatin (see above). The stoichiometry of these proteins when they assemble chromatin into LLPS condensates has typically also not tried to replicate physiological stoichiometry. For example, CBX5/HP1 forms liquid condensates in vitro that incorporate nucleosomal arrays under conditions where there is a 20–50-fold molar excess of protein [95]. Incorporating 100 µM nucleosomal arrays into a droplet forming assay reduced the critical concentration of HP1 droplet formation to approximately 150 µM in a buffer containing 75 mM KCl, 20 mM HEPES pH 7.2, and 1 mM DTT[95]. The mean concentration of nucleosomes in interphase nuclei has been measured at 110–250 µM [103, 104], while the concentration of HP1 is considerably lower, reaching maxima of about 3 µM [92]. Physiologically, then, the local stoichiometry of chromatin-binding proteins to chromatin in constitutive heterochromatin will be much lower than that required to induce LLPS in vitro. Moreover, at physiological ratios of chromatin to chromatin-binding protein in vitro, chromatin LSPS will come into play, which may fundamentally change the nature of the observed phase separation process.

Thus, conditions primarily have been used that allow LLPS of the chromatin-binding proteins to dominate while those that drive LSPS of the chromatin are weak. Under these experimental conditions, it is easy to imagine how proteinaceous liquid condensates could concentrate chromatin in vitro. The retention of KMT5C in a diffusible state within mouse chromocenters [35, 105], which contain solid-like chromatin, suggests that LLPS-driven liquid compartments rich in heterochromatin-binding proteins co-exist with LSPS-driven chromatin condensates. However, In vitro experiments analyzing the potential of LSPS chromatin condensates to nucleate LLPS of heterochromatin and the ability of the LLPS heterochromatin condensate to form in the presence solid-like chromatin are lacking.

### Euchromatin proteins

Heterochromatin domains in nuclei, e.g., chromocenters, are very distinct and relatively large structures. In contrast, transcription factors are found in small nuclear foci that can number in the hundreds per nucleus [106]. In the case of positive regulators of transcription, a phase-separated compartment is neither expected to condense nor contain high densities of chromatin. Thus, it is less clear that chromatin itself can function as a scaffold that nucleates LLPS formation by increasing the local concentration of LLPS-capable proteins. In this instance, it makes more sense that protein condensates recruit chromatin. Immunofluorescence revealed that transcription factors cluster within small nuclear foci. For example, a recent super-resolution experiment using tagged endogenous loci estimated up to 400 molecules of MED1, a member of the RNA polymerase II Mediator complex, per focus [107]. Whether that clustering reflects the underlying clustering of binding sites in chromatin or represents phase-separated liquid compartments that exist independent of chromatin is unknown. However, an additional factor may be critical in nucleating LLPS at sites of transcription—RNA. RNA is common to many types of LLPS structures that form in both the cytoplasm and nucleus [108]. There is a striking complementarity between the chromatin-rich and the RNA-rich regions of the nucleoplasm [109] and euchromatin is rich in the interface between the two [110]. Using zebrafish embryos imaged at the late blastula stage, prior to heterochromatin formation, Hilbert et al. [111] demonstrated that transcription is necessary to disperse the chromatin within the nucleoplasm. They propose that RNA polymerase II and the associated RNA serve as an amphiphile that enables the generation of microemulsions of euchromatin. In the absence of RNA polymerase initiation, these smaller euchromatic domains do not form [111].

The first links between phase separation and transcription came with experiments showing that the regulatory carboxy-terminal domain (CTD) of RNA polymerase II binds with high affinity to fibers assembled from the low complexity TAF15 protein, a substoichiometric RNA binding subunit of TFIID [112]. These domains spontaneously phase separate to form hydrogels when present in high concentrations (50–80 mg/ml) in near-physiological buffers (200 mM NaCl, 0.5 mM EDTA, 20 mM beta-mercaptoethanol). Phosphorylation of the CTD resulted in the inability to incorporate into condensates [112]. Subsequently, a histidine region found in cyclin T1, a subunit of the pTEFb kinase, and DYRK1A, another CTD kinase, was shown to form condensates with the RNA polymerase II (Pol II) CTD. The in vitro experiments revealed that the hyperphosphorylation of the CTD was inhibited at concentrations of 1,6-hexanediol sufficient to disrupt condensate formation but not direct interactions between pTEFb and the CTD [113]. 1,6-hexanediol has been used in living cells to disrupt weak hydrophobic interactions important in maintaining some phase-separated structures. While 1,6-hexanediol is widely used for melting liquid droplets formed by LLPS in vitro and in vivo, a recent report using single-nucleosome imaging revealed that 1,6-hexanediol rapidly immobilizes and condenses chromatin in living cells [114]. This action of 1,6-hexanediol is totally distinct from its droplet melting activity. Consequently, liquid droplet results obtained using 1,6-hexanediol should be carefully interpreted or reconsidered when these droplets are associated with chromatin [115]. Nonetheless, these data are consistent with the formation of Pol II CTD condensates stimulating CTD phosphorylation.

A second pair of studies looked at the Mediator complex and its interaction with RNA polymerase II [107, 116]. Med1 of the Mediator complex and BRD4, an acetylated histone binding protein enriched at super-enhancers, were found to form liquid condensates in solution, with droplets forming at concentrations less than one μM the presence of PEG8000 as a crowding agent [116]. Both the Mediator complex and RNA polymerase II were found in clusters of molecules in living cells, containing as many as 200–400 copies of each protein, and associated with transcription in pulse-labeling experiments and gene locus tracking experiments [107]. In the presence of the transcriptional inhibitor DRB, these no longer colocalize, implying an independent existence [107]. Importantly, tracking these domains revealed that they could undergo rapid fusion, but they had mobility properties similar to what has been measured for chromatin [116]. Both studies showed rapid exchange into and out of the clusters in living cells using photobleaching (FRAP) experiments and dissociation with 1,6-hexanediol [107, 116]. The condensates were also disassembled at higher salt concentrations, suggesting that the interactions are mediated by more than just weak hydrophobic interactions [116].

An important observation made in studying the relationship with pTEFb was that cyclin T1 partitions to splicing factor compartments in living cells [113]. Unlike the smaller transcription-associated clusters that form inside cells, splicing factor compartments are well-characterized by electron microscopy. They correspond to interchromatin granule clusters (see Fig. 5), which are diffuse clusters of ribonucleoprotein particles that exclude chromatin [110]. These are not sites of transcriptional engagement. A related study revealed that RNA polymerase II phosphorylation regulated a switch in RNA polymerase II partitioning between Mediator-associated condensates and splicing factor compartments. Immunofluorescent experiments show that the hypophosphorylated form of RNA polymerase II colocalized with Mediator foci but the Ser2 phosphorylated species associated with splicing factor compartments. Phosphorylation of the CTD in vitro reduced incorporation into MED1 condensates but increased incorporation into condensates formed from splicing factors (SR-repeat proteins) [117].

The organization of chromatin-binding proteins that are associated with the positive regulation of transcription and euchromatin association is strikingly similar to the organization of early S-phase replicated chromatin in size and distribution. This raises the question as to whether or not these condensates reflect an association with chromatin, similar to what is observed with heterochromatin-associated condensates, or if they have an independent existence. Using an optogenetic method to induce condensate formation, it was shown that condensates nucleated at pre-existing sites of RNA polymerase II concentration [118]. Utilizing a synthetic episomal transcription reporter containing MS2 binding sites for detection of the transcript, they further demonstrated that these sites preferentially nucleated at sites of active transcription. This argues against pre-formed condensates diffusing through the nucleoplasm to locate target genes and for the nucleated assembly in association with the target site. LLPS mediated by FET proteins can also be nucleated at the specific DNA binding site and recruit RNA polymerase II into the condensate [119]. These experiments were done using the DNA curtains assay [120], where the DNA is tethered on a slide coated with a lipid bilayer and imaged under flow conditions, stretching the DNA. Condensate assembly is analyzed by time-lapse microscopy and the extended DNA by fluorescence microscopy, enabling them to position the condensate relative to the underlying DNA sequence [119]. A chromatin-mediated nucleation process predicts that all condensates formed from proteins associated with transcriptional activation show the highly constrained diffusion behavior of chromatin.

Overall, the formation of LLPS condensates from heterochromatin and euchromatin binding proteins remains controversial [92, 115]. In vitro experiments that take greater care to reproduce the stoichiometry and physiological concentrations of divalent cations to generate solid-like chromatin condensates would provide a more relevant in vitro model for studying chromatin condensate behavior and the contribution of LLPS to the accessibility and material properties of chromatin. The principal competing model is that the concentration of chromatin-binding proteins in cells is dictated by differences in the local concentrations of the target chromatin-binding sites [92]. KMT5C [105, 121] and Rad52 [122] represent the first examples of proteins that behave as expected for proteins that diffuse within chromatin-associated compartments, heterochromatin and DNA double-strand breaks, respectively, but do not freely diffuse across the boundary between the compartment and the nucleoplasm.

### The nucleus and nuclear condensates

**Fig. 6:**
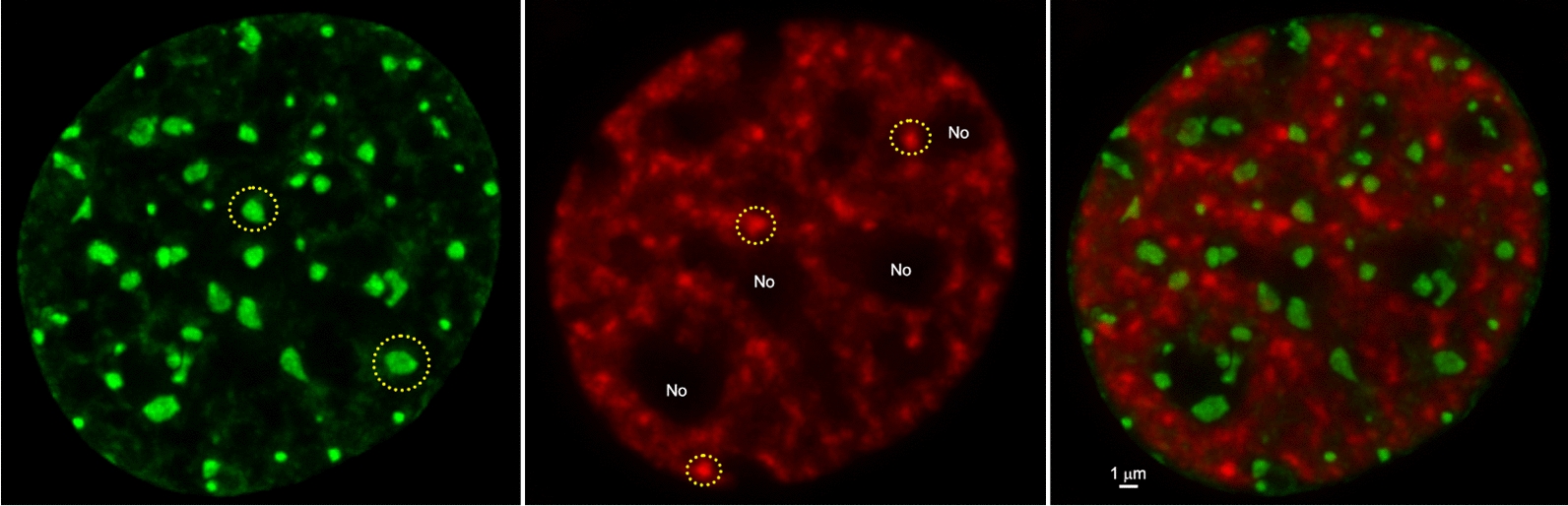
Larger nuclear compartments of the interphase nucleus. The image highlights the three large nuclear compartments present in the interphase nucleus. A living mouse C3H10T1/2 cell nucleus expressing SC35-GFP and counterstained with Hoechst 33,342 is shown following deconvolution. The SC35 (green) contrasts the splicing factor compartments and negatively contrasts the chromatin and nucleoli. The Hoechst contrasts the chromatin and negatively contrasts the splicing factor compartments and nucleoli. Circles highlight examples of large chromatin structures (pericentric heterochromatin) and splicing factor compartments in the respective images. No in the SC35 image set represents the location of the nucleoli. The scale bar represents 1 µm

**Fig. 7:**
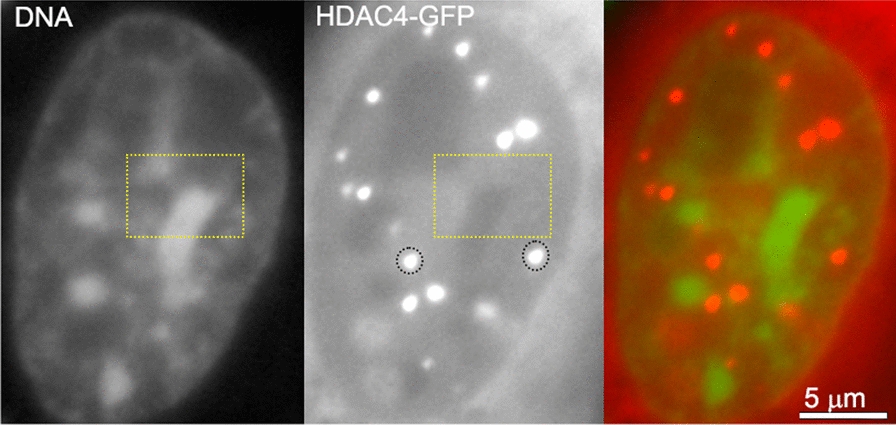
Phase-separated compartments formed from overexpression of histone deacetylase 4. SK-N-SH neuroblastoma cells were transfected with histone deacetylase 4-green fluorescent protein (HDAC4-GFP) expression vector and counterstained with Hoechst 33342 (DNA). The circles highlight condensates that form spontaneously upon overexpression of HDAC4-GFP. These are found in chromatin-poor regions of the nucleus outside of the nucleolus. The box highlights a heterochromatic region of the nucleus. Note the inverse relationship between HDAC4-GFP concentration and chromatin concentration, further highlighting that the condensates are forming outside of dense regions of chromatin. The scale bar represents 5 µm

The nucleus is a far more complex environment than what is encountered in vitro, and some understanding of its organization is essential when interpreting phase separation experiments. The protein condensates formed in vitro are typically microns in diameter. Only three structures in the typical nucleus can reach this size—heterochromatin domains, splicing factor compartments, and nucleoli (Fig. 6). Large micrometer-sized condensates that form in vivo due to overexpression of tagged proteins normally are not found within nuclei (Fig. 7). Although it is easy to demonstrate liquid-like fission and fusion events with such condensates, they are not physiological structures, and their behavior may be quite different from the smaller clusters containing these proteins when expressed at physiological levels in cells.

It is common to test liquidity using fluorescence recovery after FRAP experiments in living cells [86]. Evidence of differences in the diffusion rate of mobile proteins through the condensate or evidence of preferential movement within the condensate versus exchange with the nucleoplasm is required to show that a liquid compartment exists. Using this criterion, CBX5/HP1α and MeCP2 do not show evidence of phase separation in living cells. However, KMT5C, a histone H4 lysine 20 methyltransferase that localizes to constitutive heterochromatin, behaves as a protein trapped within a liquid compartment [105]. Notably, however, the chromatin within this compartment behaves as a solid [35].

The situation with condensates associated with transcriptional activation is much more challenging. The nucleus is relatively well-characterized by electron microscopy. Chromatin, PML bodies, splicing factor compartments, Cajal bodies, the nucleolus and nucleolar subcompartments can all be recognized in the electron microscope. There is no corresponding data on the smaller condensates associated with transcriptional regulators. One of the major challenges in defining transcription-related condensates is distinguishing them from transient binding to relatively stable chromatin. Demonstrating that molecules exchange into and out of a structure does not necessary mean that the structure is liquid in nature [86]. For example, FRAP experiments performed on transcription factors that accumulate in small nuclear foci but exchange with the nucleoplasm, one criterion of liquidity, have been interpreted and modeled as the dynamic binding of proteins to chromatin-binding sites that are immobile [123]. Criteria such as the ability to exchange across the boundary or sensitivity to 1,6-hexanediol do not distinguish between transient and direct interactions with less mobile chromatin, and the weak multivalent interactions necessary to form phase-separated liquid compartments [86, 114]. It is undeniable that positive regulators of transcription form characteristic small clusters in living cells. Whether or not the small clusters that form in cells are liquid condensates that exist independently of chromatin, are dependent on chromatin to nucleate the formation, or simply a reflection of the spatial organization of the chromatin containing their binding sites, cannot be discriminated based on the existing data. Collectively, one implication of intrinsically being able to undergo LLPS is that these proteins may be expressed at concentrations close to but below their critical concentration. This would render them sensitive to LLPS formation upon overexpression, as might occur with an expressed GFP-tagged transgene. Consequently, an inherent limitation of using fluorescent protein-tagged expression constructs to study these proteins in living cells is that they may be prone to forming LLPS condensates in the nucleoplasm if the cellular concentration is increased only slightly. This may be why larger nuclear compartments are reported in some of these experiments apart from the normal complement of splicing factor and nucleolar compartments. An example of such a result is illustrated in Fig. 7.

## Solid and liquid states of chromatin in the nucleus

Thus far, we have discussed aggregation of chromatin and specific chromatin-associated proteins into phase-separated condensates. How does the phase separation behavior of chromatin relate to the physical nature of chromatin in living cells?

### The importance of chromatin fiber self-interaction in shaping interphase chromosome structure

The chromatin fiber that makes up an interphase chromosome is a single long flexible molecule. Intuitively, the chromosomal fiber must contact itself extensively over long distances to condense into a globular territory in the nucleus. What, if any, is the relationship between self-interaction of the chromatin fiber within chromosomes in vivo and phase separation of short chromatin fragments in vitro? As discussed earlier, formation of globular chromatin condensates is driven by attractive fiber–fiber interactions and salt-dependent neutralization of DNA negative charge. The effect of salts on condensate formation is reversible. Thus, if condensates are assembled in MgCl_2_ and returned to low-salt TE (Tris–EDTA) buffer, charge repulsion overcomes the attractive nucleosome–nucleosome interactions and the condensates dissociate into dispersed nucleosomal arrays. What happens when a similar experiment is performed with isolated nuclei? Maeshima et al. [34] addressed this question by comparing the ultrastructure of isolated HeLa nuclei in buffers containing and lacking MgCl_2_. The isolated nuclei were incubated in buffer containing 0, 1, and 5 mM MgCl_2_, exposed to DAPI, and imaged by fluorescence microscopy. In 1 and 5 mM MgCl_2_, the organization of the HeLa chromatin into DAPI-dense and -faint regions, which presumably corresponds to heterochromatin and euchromatin compartments, respectively, and nuclear substructures was readily apparent as indicated by the fluctuation of fluorescence intensity across the nuclei. Strikingly, incubation of the nuclei in buffer lacking MgCl_2_ caused massive chromatin decondensation; the nuclei doubled in diameter and a halo of dispersed chromatin could be seen protruding through the nuclear surface. In addition, the nuclei seem to lose most semblance of 3D chromatin architecture as indicated by their uniform fluorescence intensity [34]. Thus, the nucleosome–nucleosome interactions that stabilize chromatin condensates in vitro and chromatin organization within the chromosome territory in situ are disrupted by electrostatic repulsion in the absence of cations. Zinchenko et al. [124] studied the salt-dependent behavior of very long nucleosomal arrays reconstituted from 165 Kb phage T4 DNA and recombinant histone octamers. These arrays contained ~ 1000 non-positioned nucleosomes per T4 DNA molecule. Results indicated that the T4 nucleosomal arrays in low salt adopted a coil structure that was converted into a compact ~ 250-nm-diameter globule in the presence of MgCl_2_. Under the same conditions, DNA did not condense. Collectively, these results imply that the same core histone tail-dependent fiber–fiber interactions that help short nucleosomal arrays assemble into globular chromatin condensates in vitro also mediate condensation of long chromatin molecules into compact globules in vitro and chromosomes in nuclei. For short nucleosomal arrays the interactions are between different arrays, whereas for long flexible nucleosomal arrays the interactions are between widely separated segments of the same array. The local and large-scale structures that result from chromatin fiber self-interaction are discussed below.

#### Local interdigitated packaging of the 10-nm chromatin fiber

The chromatin fiber in bulk in the nuclei of most eukaryotic cells exists in an extended “10 nm” conformation [62, 88, 125]. The 10-nm fiber in solution has an open zig-zag conformation (Fig. 1), which upon stretching becomes the commonly portrayed beads-on-a-string structure. For model nucleosomal arrays with homogeneous linker DNA lengths, the 10-nm fiber is a regular structure. However, chromatin in vivo has variable linker DNA lengths, nucleosome-free regions, and a specific pattern of histone post-translational modifications. As such, the chromatin fiber inside cells is more heterogeneous and irregular. Evidence suggests that the chromatin fiber interacts with itself through interdigitation, both within chromosomes and chromatin condensates. For example, SAXS analyses indicate that the packaging of chromatin found in mitotic chromosomes [38, 126, 127], intact nuclei [38], and phase-separated condensates [34] involves repetitive face-to-face and edge-to-edge nucleosome–nucleosome interactions. Interdigitated chromatin was reconstructed by a recent multi-scale computational modeling study [128] (discussed later). Under high Mg^2+^ conditions, model H1-containing dinucleosomes form crystals of interdigitated 10-nm assemblies [102]. As will be discussed further below, the propensity of the flexible chromosomal 10-nm fiber to interact with itself over long distances provides the foundation for the 3D structure of eukaryotic genomes.

#### Large-scale organization of the 10-nm chromatin fiber: the 3D genome

**Fig. 8:**
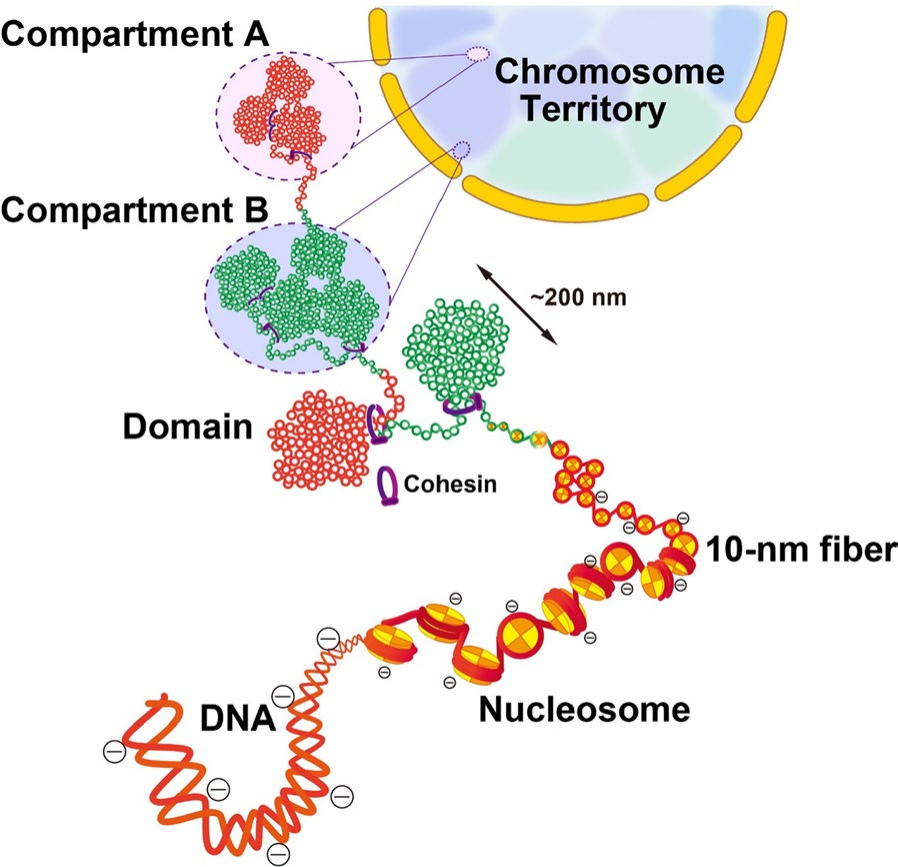
Hierarchical chromatin organization in the nucleus—a simplified view. The negatively charged 10-nm fiber is compacted into chromatin domains (e.g., topologically associating domain [TAD]/contact domain/loop domain) [136–139]. The domains are clustered over long distances to form chromatin compartments [147]. Compartments generally represent a transcriptionally active chromatin state (compartment A) and an inactive chromatin state (compartment B). A single interphase chromosome is occupied in a chromosome territory (highlighted as different colors) [219]. This illustration was reproduced with modifications from [220].

Several different lines of evidence suggest that the genomes of higher eukaryotes are partitioned into ~ 200-nm-diameter globules of densely packaged chromatin separated by regions of non-interacting chromatin (Fig. 8). Experiments in which early replicating euchromatin are labeled by incorporation of fluorescent nucleotides revealed that the labeled chromatin was organized into a punctate pattern of resolution-limited 200-nm dots [35, 129–131]. A similar punctate pattern of euchromatin organization has been visualized using fluorescently labeled antibodies against different chromatin epitopes, including the nucleosome acidic patch and histone H1.4 [132], although it is not clear whether the various antibodies label the same structures. Nozaki et al. [133] combined super resolution imaging with single nucleosome tracking in HeLa cells and observed nucleosome clustering into ~ 200-nm-diameter globular domains composed of ~ 1000 nucleosomes and ~ 200 kb of genomic DNA. Recall that reconstituted T4 nucleosomal arrays consisting of ~ 1000 nucleosome intrinsically condense into ~ 250-nm globules in vitro [124]. In the studies of Nozaki et al. [133], histone hyperacetylation caused the domains to decondense, further indicating the importance of chromatin fiber self-interaction in maintaining the compact domain structure. Bintu et al. [134] used super resolution chromatin tracing in single proliferating cells to determine the chromosomal structures present along a stretch of human chromosome 21. Results indicated that the imaged region consisted of spatially segregated ~ 200–300 nm globular domains. Recently, a combination of 3D super-resolution and scanning electron microscopy revealed chromatin domains that were composed of irregular ~ 200-nm-wide aggregates of nucleosomes [109, 135]. Taken together, the replication labeling and microscopy studies support a model for interphase chromosome organization based on globular domains of condensed chromatin. In this model, the globular structure of the chromatin domains is dictated by self-interaction of the 10-nm chromatin fiber over long distances.

Chromosome conformation capture technologies such as Hi-C reveal the regions of a chromosomal DNA molecule that are in close proximity within a folded chromosome [136–138]. The results of many such studies indicate that the chromosomes of most eukaryotes are organized into series of repetitive structures referred to as topologically associating domains (TADs) [139]. The chromatin fiber within these domains has a high probability of contact with itself compared to flanking chromatin regions, irrespective of genomic distance. Hi-C studies employing higher resolution sequencing have shown that TADs can be subdivided into ~ 185 kb “contact” domains (range of 40–3000 kb) characterized by a very high frequency of intra-domain fiber–fiber interactions [140]. Contact domains are functional units of the genome, i.e., the chromatin within the domains is mostly either transcriptionally active or repressed [140]. Although Hi-C data do not provide information about the structure of contact domains, it seems likely that they correspond to the ~ 200-nm-diameter globules of condensed chromatin observed by microscopy. Arguably the strongest evidence for this relationship comes from Bintu et al. [134] and Su et al. [141], who showed that virtually the same contact maps could be generated from Hi-C and super resolution imaging data. That is, TAD-like structures derived from Hi-C experiments corresponded closely to the spatially separated chromatin globules observed by super-resolution imaging in single cells [141].

The chromatin that surrounds TADs and contact domains is characterized by low contact frequency and often is associated with convergent CTCF/cohesin binding sites [142–144]. CTCF and cohesin assemble into structures that encircle the chromatin fiber, in the process anchoring chromatin loops (Fig. 8) that form between two convergent CTCF/cohesin binding sites. These chromatin loops frequently, but not always, correspond to the contact domains identified by Hi-C [142]. Remarkably, while cohesin loss results in the collapse of contact domains identified using Hi-C methodology [142], chromosomal domains retain their organization [134]. Fluorescence in situ hybridization experiments testing domain separation between Hi-C-defined TADs in the presence or absence of cohesin, in contrast, shows *increased* separation when cohesin is absent [145]. Collectively, these results imply that the folding into chromosomal domains occurs through self-interaction as defined above. Chromosomal domains can be independently folded regions of the genome that coincide with Hi-C-defined TADs. Interestingly, cohesin loss only alters the expression of a subset of genes but those that show the greatest loss of expression correlate with genes that are close to domain boundaries and with genes in large domains [146]. Thus, a role of cohesin/CTCF is to promote long-range self-interaction of the chromatin fiber that occurs within the ~ 200-nm-diameter chromosomal domains.

Beyond the level of ~ 200-nm chromatin globules, Hi-C experiments have revealed the existence of two “compartments”, termed A and B [147]. Functionally, compartment A most closely corresponds to euchromatin and compartment B to heterochromatin although this is a simplification. A and B compartments both can be subdivided into several sub-compartments as defined by their specific chromatin signatures [142]. The chief physical characteristic of compartments is that they preferentially interact with other regions of a chromosome that have the same chromatin signatures, leading to a checkerboard pattern on the Hi-C contact maps [147]. In other words, the chromatin of A compartments preferentially interacts with itself and not the chromatin of B compartments and vice-versa.

The microscopy and Hi-C data can be combined into model of genome organization suggesting a hierarchical relationship between ~ 200-nm globular domains and compartments (Fig. 8). In these models, each ~ 200-nm chromatin domain consists primarily of either active or inactive chromatin as defined by its pattern of post-translational modifications, the nucleosome spacing and arrangements within the globule, and the specific cohort of proteins present. The boundaries of most ~ 200-nm chromatin domains are defined by CTCF/cohesin, which demarcate the chromosome into functional units. Interaction of ~ 200-nm globules with other domains sharing the same chromatin signature establishes A and B compartments and their subcompartments. All steps in the hierarchy are fundamentally driven by core histone tail-mediated self-interaction of the chromatin fiber. The selectivity of domain–domain interactions to form A or B compartments may be mediated by interactions between specific chromatin-associated proteins in the domains that are superimposed upon the intrinsic histone-mediated chromatin fiber interactions.

### Condensed chromatin in the nucleus behaves like a solid on the mesoscale

Several early kinetic experiments are suggestive of solid-like behavior to chromatin under normal conditions. FRAP experiments done on core histones revealed very slow recovery [148–150]. For example, the T_1/2_ of the slow recovering population of H2B was greater than 510 min and the recovery of the slow population of H3 and H4 could not be determined (essentially immobile) [148]. Even in early embryonic development, where the chromatin is in a more “open” conformation, and there is a higher proportion of the core histone pool that is mobile, the majority of the pool is “stable” during the experiment [151]. The persistence of photobleached chromatin over hours demonstrates the failure of the chromatin to mix. The ability to fix this chromatin in space and the failure of the chromatin to recover by diffusion-mediation invasion of labeled chromatin into regions occupied by photobleached chromatin provided indirect evidence that the chromatin at the chromosome scale is not undergoing mixing that is characteristic of liquids. Similarly, tracking photo-activated histones across the cell cycle revealed some increased fuzziness to the photo-activated chromatin but the overall organization was well maintained over hours [152].

The principal limitation of these experiments is the interpretation of movement of fluorescent histones into photobleached regions as a measure of liquid-like behavior [86]. Because histones can be displaced from chromatin, there is some recovery, and we cannot rule out that some of this is due to mixing of the fluorescent and non-fluorescent chromatin rather than the exchange of the histone. The stability of fluorescent nucleotides incorporated into chromatin enabled Strickfaden et al. [35] to address the physical properties of chromatin in living cells using FRAP. Using this approach, the fluorescence did not recover within the photobleached region when either early S-phase replicated euchromatin domains were photobleached or later replicating heterochromatin regions were photobleached. These experiments revealed that there was no resolvable mixing of chromatin within heterochromatin but could not resolve whether or not there is mixing within euchromatin. Rather, the behavior of euchromatin in FRAP experiments is consistent with there being no mixing between chromosomal domains but it does not tell us about mixing within these domains. There are other important limitations to these experiments. The nuclei were not experiencing significant changes in applied forces from the cytoskeleton that could reveal further information on the material properties of the chromatin. The response of chromatin under applied forces may be different. The FRAP results do not distinguish between a very viscous liquid, a viscoelastic gel, or a rigid solid. However, they do demonstrate that over at least tens of minutes, in the absence of such stresses, chromatin does not mix.

Although chromatin has solid-like properties when it is not under strain and we are measuring the mixing of chromatin fibers within chromatin condensates found in living cells, this does not tell us much about how the chromatin behaves under conditions where forces are exerted on the nucleus. Direct measurements of chromatin in the presence of applied forces, endogenous or exogenous in origin, can reveal other properties that inform us on the behavior of chromatin, such as elasticity and viscosity. The response of mitotic chromosomes to mitotic spindle tension provided one of the first clear examples of chromatin having elastic properties, consistent with solid-like behavior. Bouck and Bloom [153] examined the separation of sister kinetochores and demonstrated, through histone depletion, that pericentric heterochromatin has elastic behavior in the presence of kinetochore tension.

To study interphase chromatin, Shimamoto et al. [154] used two microneedles to puncture isolated HeLa nuclei and then, by separating the needles, apply force to the nuclei. Resistance to the force was measured by displacement of the force-calibrated microneedle. These experiments revealed that nuclease digestion of linker DNA reduced the rigidity of the nucleus approximately threefold and that histone hyperacetylation reduced the resistance to the applied force approximately twofold [154]. Consistent with the impact of magnesium ions on chromatin condensate formation in vitro, nuclei had greater stiffness in the presence of 5 mM MgCl_2_ versus 1 mM. A comparable response to magnesium concentration on the stiffness of nuclei was observed by Stephens et al. [155] using a very similar microneedle approach in isolated murine embryonic fibroblasts. They also found that histone acetylation reduces nuclear rigidity and further uncoupled the contribution of the nuclear lamina from chromatin, concluding that chromatin is the dominant resistant force to extension less than three micrometers [155]. Similar conclusions were reached by Wintner et al. [156].

A challenge in studying the contribution of chromatin to the mechanical properties of the interphase nucleus is that the nuclear lamina forms an elastic shell at the nuclear surface that is clearly important in the mechanical integrity of the interphase nucleus. This is further complicated by reduced chromatin condensation in lamin mutant cell lines and evidence that the two may be normally interdependent [156]. Notably, lamin A-depleted cells have reduced perinuclear heterochromatin [156]. Comparing the response of lamin A, B1, and B2 knockouts, lamin A knockouts, and wild-type cells, they were able to attribute viscous and elastic force contributions of chromatin and each lamin. Triple knockout cells are soft and chromatin decondensation does not result in any further softening. Lamin A knockout cells are stiff but less viscous and chromatin decondensation softens the nuclei [156]. The contribution of chromatin to the stiffness of nuclei was also evaluated in *S. pombe* wild type and mutant cells in which the tethering of chromatin to the nuclear envelope was disrupted [157]. In the absence of tethers, the nuclei were softer and were more prone to responding to forces through chromatin movement (flow). In wild-type cells, chromatin contributed to the elastic response of the nucleus and the nuclei were stiffer. Consistent with an important role for histone N-termini in contributing to this response, upon histone hyperacetylation by treatment with Trichostatin A, both wild type and mutant cell lines were significantly softer [157].

There is emerging evidence that histone post-translational modifications influence the mechanical properties of the nucleus and that cells modulate their epigenetic state to adapt to changes in applied stress. Using an auxin-inducible degron system to deplete cellular CBX5/HP1α, Strom et al. [158, 159] found that loss of CBX5/HP1α results in significant nuclear softening but not the loss of heterochromatin domains, consistent with their persistence when the methyltransferases responsible for synthesizing H3K9me3 are knocked out [160]. HP1 can dimerize and potentially cross-link chromatin fibers together [161]. The decrease in the hardness of the nuclei in the absence of HP1 [159] suggests that the HP1-dependent interactions are stronger when challenged by applied forces than the intrinsic fiber–fiber interactions mediated by the histone N-termini. CBX5, as an HP1 family member, recognizes trimethylation of histone H3 lysine 9. This modification is abundant in pericentric heterochromatin. Two recent studies have found that this methylation is regulated when cells experience prolonged exposure to applied force [162, 163]. Stretching experiments done on epithelial progenitor cells found that they have an initial response of downregulating H3K9 trimethylation in a calcium-dependent manner preceding a longer adaptive response involving reorganization of the cytoskeleton [162]. This loss of H3K9 trimethylation is required to reduce the stiffness of the nucleus and failure to do so results in DNA damage [162]. Interestingly, in murine embryonic fibroblasts, the opposite response was seen. When mechanosensitive ion channels were stimulated by adding divalent ions or polyamines to the media, these cells upregulated histone H3 lysine 9 trimethylation and increased their stiffness [164]. These results demonstrate that cells adapt the mechanical properties of chromatin in response to the demands of their environment. The physical models of chromatin mesoscale organization need to be able to account for the contribution of chromatin to the mechanical stability of the nucleus and the manner in which the nucleus responds to applied forces. Physiologically, cells experience such forces. In the case of cardiomyocytes, for example, they undergo significant deformations of their nucleus rhythmically as the cells contract and relax during a heartbeat [165].

### Chromatin mobility on the nanoscale

**Fig. 9:**
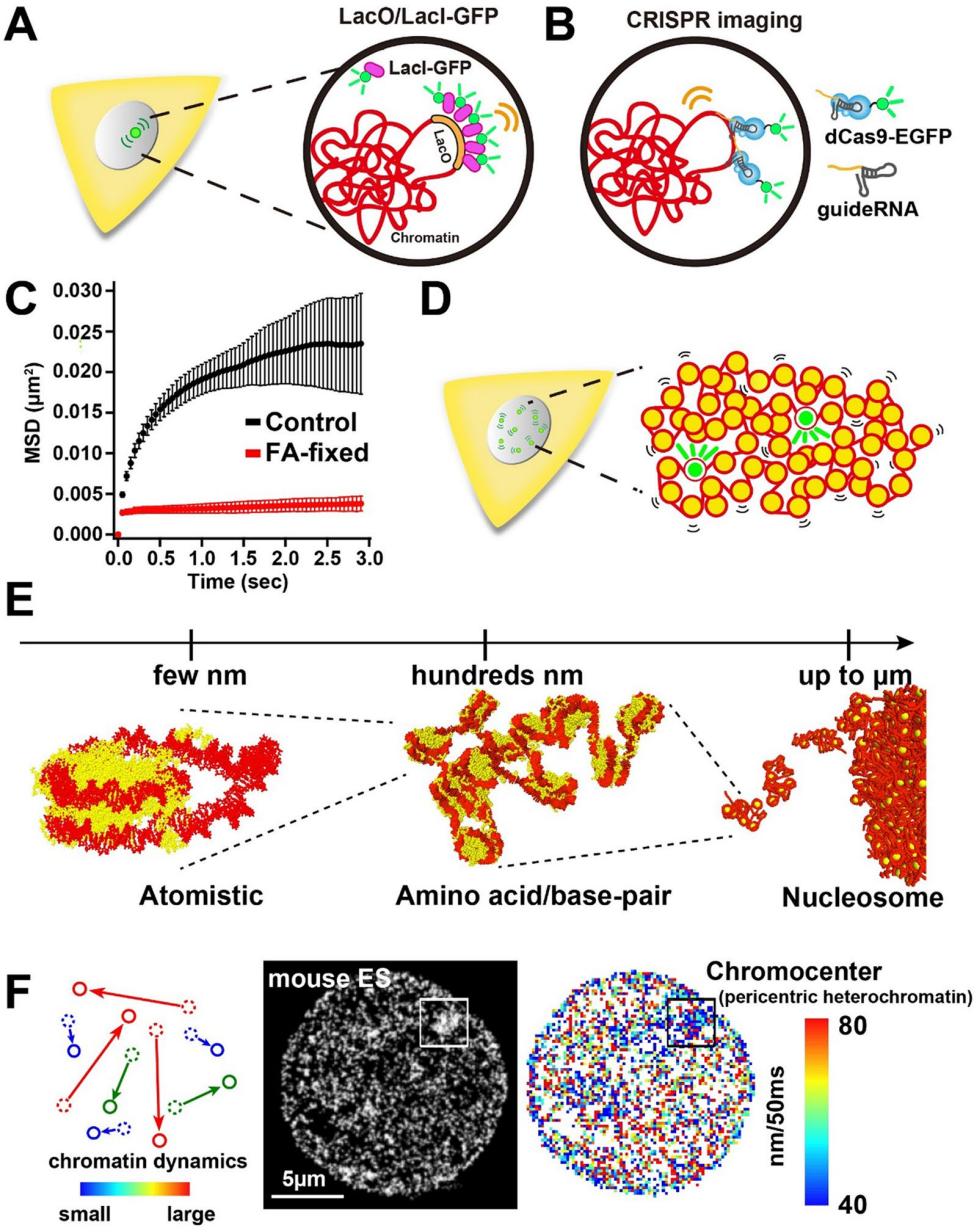
Visualization of dynamic chromatin motion. Schemes for LacO/LacI-GFP (**A**) and CRISPR-based chromatin labeling (**B**). **C** Constrained diffusion motion of chromatin: mean square displacement (MSD) plots (± SD among cells) of single nucleosomes in living (black) and formaldehyde-fixed (red) human RPE-1 cells over time (0.05 to 3 s) (data from [185]). **D** Scheme for single-nucleosome tracking. A small number of nucleosomes are labeled with photoactivatable GFP or other fluorescent tag to get sparse labeling. **E** Multiscale model of chromatin integrating three resolutions: atomistic (left), amino acid/base pair (center), and nucleosome (right) [128]. These models allow the exploration into how atomistic and residue level variations affect the structure and dynamics of chromatin fibers and domains. Illustration was reproduced from [189]. **F** (left) Scheme of chromatin heat map. In the heat map, small movements for 50 ms are shown in blue, and large movements are shown in red. (center) PALM (photo-activated localization microscopy) super resolution image and the chromatin heat map of a living mouse ES (embryonic stem) cell (right). Heterochromatin regions (nuclear periphery and pericentromeric heterochromatin) show dark blue. Data reproduced from [133]

Chromatin mobility in the nucleus on the nanoscale was initially documented with conventional epifluorescence time-lapse microscopy. The LacO/LacI-GFP system [166] (Fig. 9A) was the first system employed to discover and characterize the dynamic movements of chromosomal loci in organisms such as yeast, nematodes, flies, and mammals [167–170] [171–173]. A related system was also developed [174]. Recently, genome editing technology with CRISPR/Cas9 or CRISPR/dCas9 has allowed labeling and visualization of specific genomic chromatin regions (e.g., [175]) (Fig. 9B) and confirmed their subdiffusive motions [176–178]. Free diffusion is linear with time. Subdiffusive motion reflects motion where diffusion is constrained in some manner and distance does not increase linearly with time (Fig. 9C). In locus tracking experiments, diffusion is constrained since loci primarily move around in a small volume. A complication of these experiments is that there is no correction for large-scale coordinated movements of chromatin that have been observed in living cells on seconds to minutes time-scales. These studies implemented corrections for nuclear rotation but not intranuclear movement. Because the nucleus is subjected to forces applied by the cytoplasm that can rotate it in x, y, and z dimensions and local forces that impact only regions of the nucleus, it is important to consider these as potential contributors to reported motions even when attempts are made to correct for them.

It should be noted that not all locus tracking experiments have revealed exclusively random diffusive motion. Instances of directed movements of individual loci have been demonstrated and not all instances of loci tracking reveal strictly random motions. For example, Dundr et al. [179] incorporated an tetracycline inducible array of U2 snRNA genes containing LacO repeats to show a transcription- and actin-dependent relocation of the array to Cajal bodies over a distance of 1.5 to 3.0 microns. Similar actin-dependent vectoral transport was observed upon transcriptional activation for an array containing an HSP72 construct translocating from the nuclear periphery to splicing factor compartments [180]. More recently, Wang and colleagues used yeast cells to investigate the translocation of the INO1 gene upon inositol induction. They demonstrated that the translocation requires the Arp subunits of the Ino80 chromatin remodeling complex, actin polymerization, and the actin binding protein formin [181]. Thus, chromatin can be moved intranuclearly through both active and passive mechanisms, rearranging chromosomal domains relative to each other in space.

In addition to single-locus tracking, fluorescence correlation methods have been used to extract diffusion and movement properties of many independent regions of chromatin simultaneously. Early S-phase replicated foci and histone H2B-GFP have been used to examine the diffusion of chromatin in living cells. This has confirmed that constrained diffusion predominates at short time-scales. However, over the course of seconds and persisting for minutes, coordinated movement (flow) of patches of chromatin as large as 5 µm in diameter is observed [182, 183]. Moreover, the linking chromatin between the individual early S-phase replicated domains can be inferred to be flexible based on the dynamics of chromatin labeled with fluorescent nucleotides early in S-phase [184]. Tracking multiple chromosomal sites, Ma et al. [176–178] found that these radii of diffusion varied with cell cycle, with maximal freedom of movement in late G2 and early S-phase, but that all loci were confined to spheres with radii of between 0.02 and 0.3 microns. While differences in the absolute radius and rates of diffusion showed some variation in magnitude, the general conclusion was that any region of chromatin will undergo constrained diffusion due to thermal fluctuation. However, this type of mobility does not inform us on the mixing of nucleosomes or individual arrays of nucleosomes within each 200-nm domain. For example, the constrained diffusion may be explained by individual 200-nm domains in a gel state connected by flexible intervening chromatin. To determine whether or not the 200-nm chromatin domains are liquid-like condensates, it is necessary to understand the movement of nucleosomal arrays relative to each other within these domains. This currently is only feasible using single-molecule tracking approaches.

The LacO/LacI and CRISPR studies examined the movements of relatively large segments of chromatin (~ 20–50 nucleosomes), raising the question of what kind of motions individual nucleosomes undergo. To address this issue, Hihara et al. [104] performed single nucleosome imaging experiments in live cells. Histone H4 was fused to photoactivateable GFP and used to label single nucleosomes throughout the genome (Fig. 9D). Nucleosome movements were then visualized and quantitated using fluorescence microscopy. The average displacement of a labeled nucleosome in interphase chromatin over a 30-ms interval was ~ 50 nm, confirming nanoscale motions of chromatin. Nozaki et al. [133] combined super-resolution PALM imaging with single nucleosome tracking using photoactivatable mCherry-H2B (Fig. 9D). They found that nucleosomes moved ~ 60 nm in 50 ms, consistent with previous measurements [104]. Analysis of super-resolution PALM imaging revealed that nucleosomes clustered into globular 200-nm domains (described above). Moreover, evidence was obtained for coherent diffusion of the nucleosomes and the domains they occupied [133]. Thus, at least part of the nucleosome mobility seen in the single nucleosome tracking experiments results from the movement of the higher order domains they are found in. Nagashima et al. [185] and Lerner et al. [185] also observed local nucleosome motions genome-wide with single nucleosome imaging, in this case using HaloTagged H2B. Rapid local chromatin movements has also been obtained by combining confocal microscopy of fluorescently labeled bulk histones or DNA binding fluorophores with displacement correlation analysis [182, 186]. Finally, statistical analyses [187, 188] of genome-wide single nucleosome tracking data [133] have demonstrated that the genomic nucleosomes show fluid-like behavior at the 300-nm length scale. Taken together, these studies demonstrate that chromatin locally is not a rigid solid and has some dynamic features that are suggestive of liquid-like properties. These properties are consistent with the findings that genomic chromatin consists of irregular extended structures in which nucleosomes are interdigitated or clustered together (discussed above).

Furthermore, this liquid-like behavior of chromatin was reconstructed by a recent multi-scale computational modeling [128, 189]. Atomistic simulations allow the investigation of the nucleosome and di-nucleosome systems, reaching system sizes of a few nanometers (Fig. 9E). To simulate chromatin, coarse-graining is required. Farr et al. use a ‘chemically specific’ model (representing histone proteins at the level of one bead per amino acid and DNA at base-pair resolution) to model chromatin fibers [128]. Investigating the collective behavior of chromatin domains requires a further reduction in the number of particles representing a nucleosome, giving a nucleosome resolution ‘minimal’ model. Combined, these models allow the exploration into how atomistic and residue level variations affect the structure and dynamics of chromatin fibers and domains. Multiscale modeling of 12-nucleosome arrays revealed that nucleosome breathing facilitates chromatin phase separation: when nucleosomes can breathe, liquid droplet condensates begin to appear at lower concentrations of salt because breathing nucleosomes foster more connections [128, 190].

Local chromatin motion seems to be isotropic and primarily driven by thermal fluctuation [133, 167, 170, 171, 188]. On the other hand, as discussed above, ATP-dependent directional chromosomal motions, which often correlate with RNA transcription or DNA double-strand break repair have been reported (see recent reviews [191, 192]), although the forces responsible need further investigation. Local nucleosome motion within the nucleus is heterogeneous. Nozaki et al. [133] displayed their single nucleosome tracking data as a heatmap, which revealed that nucleosomes near the nuclear periphery are less mobile than interior nucleosomes (Fig. 9F). Statistical analysis of the same nucleosome tracking data identified fast and slow populations of mobile nucleosomes, with the slower nucleosomes present at the nuclear periphery [187, 188]. Chubb et al. [167] used the lacO/LacI system to investigate the relationship between locus mobility and nuclear location. They observed that loci associated with the nuclear periphery and the nucleolar surface have reduced mobility compared to those that localize to the nuclear interior. These results imply that the local motion of or within constitutive heterochromatin are more constrained than those in euchromatin and that association with the nuclear lamina or nucleolus may further constrain that motion.

Importantly, evidence for the functional significance of local chromatin motions has been obtained [133, 181, 193–195]. The low mobility chromatin was enriched at the nuclear periphery while the high mobility chromatin was more prevalent in the interior [193]. In addition, Lerner et al. [196] showed that heterochromatin proteins (e.g., HP1, Suv39h1) are preferentially located in the low mobility chromatin fraction, solidifying the connection between constitutive heterochromatin and slower local nucleosome motion. This may be due in part to the ability of HP1 to cross-link adjacent nucleosomes in the chromatin fiber [197], thereby constraining local motion. Lerner et al. [196] also determined how various transcription factors partitioned between the different chromatin mobility groups. Strikingly, the pioneer factors FOXA1, SOX2, OCT4, KLF4, and PU.1, which bind nucleosomes strongly, were enriched in low and very low mobility chromatin. Some of these pioneer factors were present in high mobility chromatin while others were excluded. On the other hand, cMYC and GATA4, which bind nucleosomes more weakly, were depleted in low mobility chromatin and enriched in high mobility chromatin. HNF1A and 4A are differentiation factors that do not bind nucleosomes, and these proteins were essentially absent from low mobility chromatin and only found in high mobility chromatin. The observation that nucleosome motions in part result from movement of the 200-nm domains they are found in [133] raises the question of whether the different chromatin mobility groups identified by Lerner et al. [196] reflect different subtypes of 200-nm domains that vary in their chromatin landscape and functional activity, as implied by the work of Rao et al. [140].

**Fig. 10:**
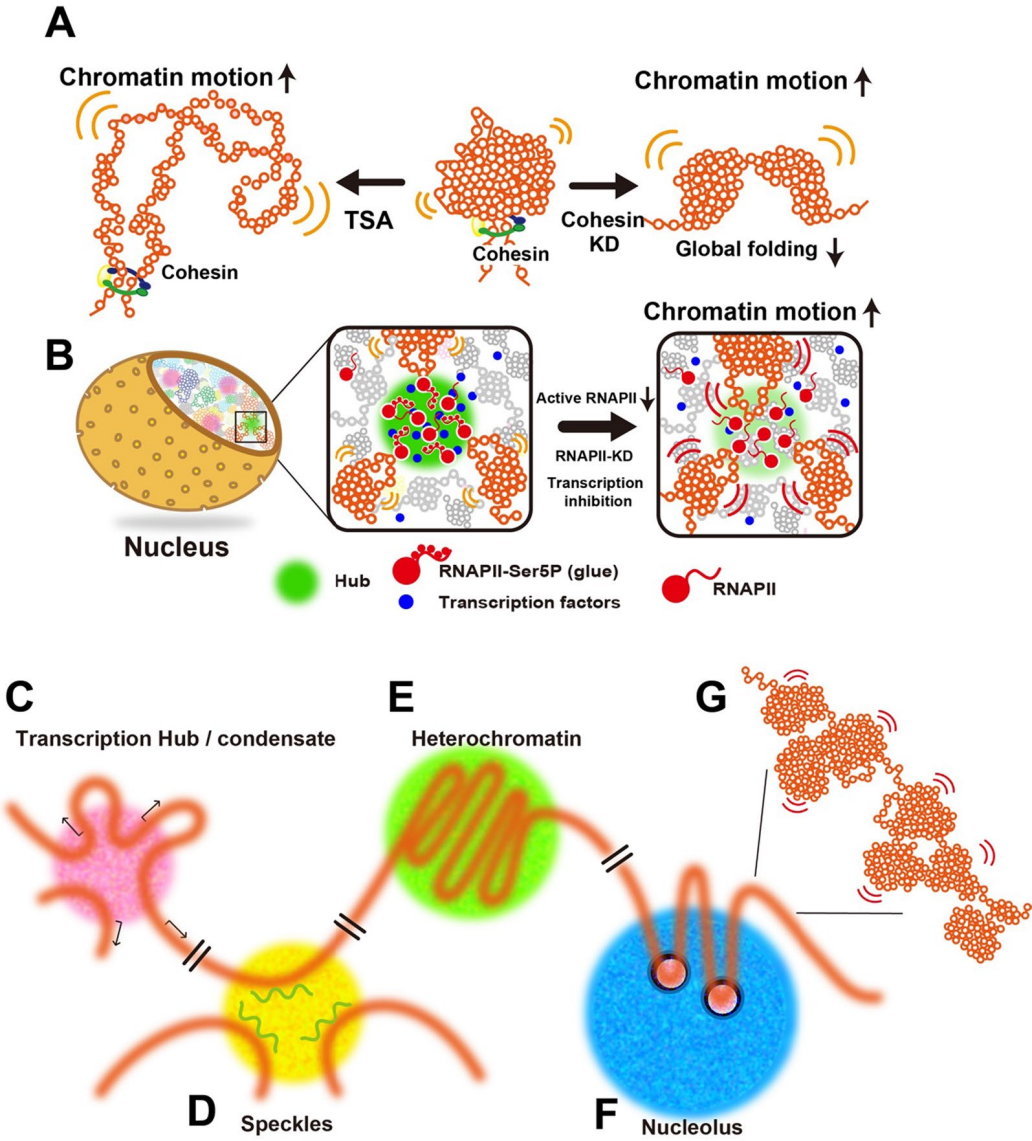
Local chromatin motion in the cell. **A** (left) Decondensed chromatin in cells treated with histone deacetylase (HDAC) inhibitor TSA showed increased chromatin movements because of weakening nucleosome–nucleosome interactions and subsequently less local chromatin constraint. (center) The typical state of chromatin: chromatin domains are organized by local nucleosome–nucleosome interactions and global folding by cohesin. (right) Cohesin loss leads to less constraint and a resultant increase in chromatin motion. **B** (left and center) Cluster/condensate of active RNAPII and transcription factors (blue sphere) can work as a transient hub (green sphere) to weakly connect multiple chromatin domains and to globally constrain chromatin motion. (right) RNAPII inhibition or its rapid depletion releases the chromatin constraints and increases chromatin motion. **C**–**F** Cartoons showing how various nuclear condensates are organized with different chromatin substrates. The orange line represents chromatin. Chromatin seems solid at the mesoscale and behaves like a liquid at the nanoscale, which is consistent with its viscoelastic property [216] (**G**). Black arrow designates active transcription start site, and green squiggled lines show RNA. Schemes in Panels A and B were reproduced from [220] with modifications

While chromatin is in motion and 200-nm domains undergo movements by thermal fluctuation [187, 188, 192, 193, 196], as discussed above, chromatin behaves more as a type of solid on the mesoscale. This is obvious at the chromosome level; interphase chromosomes form discrete territories in the nucleus rather than mix their content based on, for example, functional relationships (see [198] for a review). What restricts the motion of genomic chromatin over long distances? Perhaps most fundamentally, nucleosome motions are constrained by self-interaction of the chromatin fiber. Recall that chromatin self-interaction is mediated by histone tail–DNA interactions and that only a fraction of the tails are needed to induce chromatin LSPS in vitro [40, 193]. Presuming that the latter observation applies to chromatin fiber interactions in vivo, one can envision a model in which long-range tail–DNA interactions will cross-link genomic chromatin, creating a solid-like environment globally. Importantly, in this model, many of the nucleosomes within a self-interacting chromatin region will not participate in cross-linking interactions, allowing for local fluctuating motions. In vivo evidence for the involvement of tail–DNA interactions comes from histone acetylation studies. It has been known for many years that HDAC inhibitor-induced hyperacetylation of the histone tails is correlated with increased sensitivity of genomic chromatin to nuclease digestion, an indirect readout of the extent of global chromatin condensation and fiber–fiber self-interaction [199]. TSA-induced chromatin decondensation in living cells has been observed by PALM imaging [35, 133, 200, 201]. Notably, the nucleosome tracking experiments and subsequent statistical analyses of the tracking data showed that treatment of HeLa cells with TSA led to increased local nucleosome motions throughout the nucleus [133, 188, 202] (Fig. 10A). Mechanistically, hyperacetylation significantly weakens tail–DNA interactions [46] and reduces the extent of DNA charge neutralization [47], both of which would be expected to enhance local nucleosome movements. It is notable, in this respect, that histone acetylation, which reduces chromatin fiber interactions, also reduces the ability of nuclei to resist applied force [154]. This implies that long-range tail–DNA interactions promote a more rigid state.

Mg^2+^ promotes chromatin phase separation in vitro as well as maintains chromosomal integrity in isolated nuclei [34, 34]. These observations suggest that the extent of chromatin condensation, and subsequently the local chromatin motions, may be regulated by intracellular Mg^2+^ levels. This possibility recently has been investigated. In their single nucleosome tracking experiments, Nozaki et al. [133] observed that a decrease in nuclear ATP caused a decrease in local nucleosome mobility. Given that ATP in the cell exists as a complex with Mg^2+^, one explanation for this result is that ATP hydrolysis releases free Mg^2+^, which then goes on to modulate local and global chromatin structure. To test this hypothesis, Maeshima et al. [63] developed a novel FRET-based Mg^2+^ indicator capable of measuring free Mg^2+^ levels in the nucleus. Using this reagent, they showed that free Mg^2+^ becomes elevated during mitosis concomitant with hypercondensation of mitotic chromosomes. Decreased ATP levels also enhanced mitotic chromosome condensation. Thus, it appears that the ATP-dependent decrease in local nucleosome movements observed by Nozaki et al. [133] results from Mg^2+^-dependent increases in chromatin condensation. It should be noted that there was no marked changes in free Mg^2+^ during interphase [63]. However, the data were noisy, and it is possible that there are transient fluctuations in Mg^2+^ levels throughout the cell cycle that locally regulate chromatin condensation. Altogether, these findings suggest a novel regulatory mechanism for modulating the extent of chromatin fiber self-interaction (i.e., condensation) and local nucleosome movements by the intracellular Mg^2+^-ATP balance. In this regard, hypertonic treatment (~ 570 mOsm) of cells, which increases intracellular cations and molecular crowding [203], also increases chromatin condensation and decreases local nucleosome motions [35, 133].

Various chromatin-associated proteins constrain local chromatin motion driven by thermal fluctuation. The cohesin complex captures chromatin fibers with its ring structure to form loops of condensed chromatin during interphase and attaches sister chromatids together during mitosis [204–206]. In the case of loop domain formation, cohesin is present at the base of the loop. siRNA-mediated depletion of the cohesin RAD21 subunit together with single nucleosome tracking showed that disruption of the cohesin complex caused more uniform distribution of the labeled nucleosomes throughout the nucleus, led to chromatin decondensation, and increased local nucleosome mobility (Fig. 10A) [133, 188, 194]. It seems likely that other chromatin-associated structural proteins will influence nucleosome mobility as well. For example, linker histones reduce the mobility of chromatin in vitro in phase-separated condensates [33, 35], suggesting they also may modulate local nucleosome mobility in a chromosomal environment in the nucleus.

The transcriptional machinery has a constraining role for local chromatin motion (Fig. 10B, C). Germier et al. [174] used the ANCHOR DNA labeling system to fluorescently label a single gene locus and showed that transcription initiation leads to confinement of the gene. Chen et al. [207] found that transcriptional activation led to reduced local movement of a functional enhancer–promoter pair. At the single-nucleosome level, Nagashima et al. [185] used the same H2B-Halo approach as in [133] to investigate the role of Pol II transcription in modulating local nucleosome mobility genome-wide. Treatment of cells with the Pol II inhibitors, α-amanitin and DRB, in each case led to significant increases in local nucleosome movement [185]. Both of these inhibitors reduce the amount of Pol II bound to chromatin, suggesting that Pol II constrains the local chromatin environment. In support of this conclusion, treatment of cells with Actinomycin D, which stalls Pol II and leads to its accumulation in chromatin, caused decreased local chromatin mobility. Nagashima et al. [185] further found that auxin-dependent degradation of Pol II, serum starvation, and exposure to UV radiation all increased local chromatin motions. Similarly, image correlation methods that analyze chromatin motion nucleus-wide have also demonstrated transcription-dependent reduction in mobility [182, 208]. Taken together, these results demonstrate that active Pol II restricts local nucleosome movements. Increased local nucleosome motion also was induced by knockdown of TEFb, which phosphorylates the Pol II CTD on serine 2 and promotes transcription elongation. Based on these results, Nagashima et al. [185] proposed a model in which euchromatic 200-nm domains form a loose network held together by hubs consisting of Pol II, transcription factors, and regulatory proteins such as P-TEFb (Fig. 10C). These hubs correspond to the small foci discussed in the second part of the review, which may arise from LLPS of their components [107, 113, 116, 209]. This model is an extension of the transcription factory model first proposed a number of years ago [210, 211].

An analogous situation appears to exist for RNA polymerase I (Pol I) in the nucleolus. Ide et al. [212] labeled Pol I with HaloTag and tracked its diffusion within the fibrillar center (FC) of the nucleolus. The data showed subdiffusive properties, indicating that the movement of Pol I was constrained. To determine the movements of the rDNA chromatin within the FC, HaloTagged upstream binding factor (UBF) was tracked. Plots of mean square displacement against time for Pol I and UBF were almost identical. Pol I formed clusters on rDNA chromatin, leading Ide et al. [212] to propose that clustered Pol I acts as glue to constrain the movement rDNA chromatin in the FC. In support, transcription inhibition caused Pol I to dissociate from rDNA chromatin and removed the constraint to both Pol I and UBF movement in the nucleolus. Thus, the transcription factory model [210, 211] also may apply to Pol I. It will be interesting to determine whether polymerase and transcription factor condensates can organize the surrounding genomic chromatin into specific functional structures.

As discussed above, the nucleosomes within heterochromatin domains are less mobile than those in euchromatin (Fig. 9F) [133, 167, 185, 196, 213]. At least part of this reduced nucleosome mobility can be traced to the structural effects of the HP1 proteins, which selectively bind to nucleosomes marked with H3K9me2/3. These modifications are most prominent in the constitutive heterochromatin domains found at the nuclear periphery such as lamina-associated domains [214], as well as locations scattered throughout the nucleus. HP1 family proteins have a chromodomain (CD) and a chromoshadow (CSD) domain connected by hinge region. HP1 forms a dimer through its CSD domain. The HP1 dimer can further self-associate into higher order oligomers, including tetramers and octamers [161]. HP1 binds to an isolated H3K9me3 mononucleosome as a tetramer [161]. However, a fundamentally different HP1 binding mode is suggested by the recent cryo-EM structure of HP1 bound to a H3K9me3 dinucleosome. In this case, a single HP1 dimer physically contacts both nucleosomes of the dinucleosome, creating a rigid bridge between them [197]. If HP1 binds to the chromatin fiber in vivo in this fashion, it would be expected to stiffen the chromatin fiber and significantly reduce local nucleosome mobility within heterochromatin domains. Chromatin motion can be further restricted within heterochromatin domains by long-range cross-linking of the chromatin fiber by HP1 dimers [193]. Strom et al. demonstrated that HP1 and specifically HP1 dimerization is not required for heterochromatin condensate maintenance but, through its cross-linking function, contributes to the rigidity of the nucleus [158]. It should be noted that the cross-linking function of HP1 appears to be independent of its ability to phase separate [92]. Finally, at the nuclear periphery, HP1 may work together with inner nuclear membrane proteins such as lamins to constrain chromatin movement [133, 167, 185, 196, 213].

## Concluding remarks

We have so far discussed two physical states of chromatin: liquid-like or solid-like. To distinguish a liquid or solid largely depends on the time and dimension scales. As shown in the famous pitch drop experiment, which is well-known as one of the longest-term experiments [215], some substances that appear solid to our vision are actually highly viscous liquids. Such substances flow at a very low rate, taking several years to form a single droplet. Thus, the question of liquid versus solid chromatin is a matter of perspective. Here we have defined solid chromatin as that which does not mix with its environment on the minutes to hours timescale. This is in contrast to the constrained local motion of nucleosomes that occurs on the seconds timescale. It should be noted that substances that have both solid-like and liquid-like properties, depending on the time scale, are referred to as viscoelastic [133, 216–218]. As discussed throughout this review and also by Erdel [216], this definition fits the properties of condensed chromatin.

The correlated movement of chromatin domains on longer time-scales and the local motion on shorter time-scales correlating with the movement of single nucleosomes implies that most of the motion studied to date may be explained by chromatin forming gel condensates of approximately 200-nm diameter that are connected by flexible linkers of chromatin that are resistant to cross-linking. As we have discussed, it is tempting to speculate that the 200-nm chromatin domains might be liquid-like. These are genomic locations where Hi-C maps demonstrate increased frequency of interactions across the domain, suggesting that the chromatin is continually mixing, as expected of a liquid. A liquid state to this chromatin, mediated by reduced tail–DNA interactions, would enable the efficient activation of genes through diffusion-mediated long-range regulatory contacts. However, recent Hi-C and super-resolution microscopy experiments have revealed that the 200-nm domains seem to be maintained, show less spatial overlap, and persist in the absence of cohesin [109, 145]. This indicates that these domains are not held together as a unit through cohesin-mediated boundaries. The reduction in long-range regulatory interactions, the loss of elevated frequencies of long-range Hi-C contacts within TADs, and the increased spatial overlap between domains in the presence of cohesin is consistent with the cohesin activity playing an important role in mediating long-range interactions. This suggests that the 200-nm domains also may persist in a solid gel state, requiring cohesin for efficient relocation of regulatory sequences relative to each other.

Our current understanding of the liquid and solid states of chromatin is largely at the level of empirical observation. We are just now starting to appreciate the dual nature of chromatin, both in vitro and in vivo, and the biological reasons why chromatin is this way still need to be uncovered. Many questions remain. Is the reason why interphase chromosomes exist as territories because each chromosome fundamentally is a solid chromatin condensate that has phase separated from the nucleoplasm? At what length scale does the transition from liquid chromatin to solid chromatin occur? Is it gradual or cooperative? Can a specific region of solid chromatin be converted to liquid chromatin by extraneous factors in the nucleus? How much of the observed motion of single nucleosomes is due to movement of the 200-nm domains that the nucleosomes occupy? Do liquid compartments of heterochromatin proteins require solid condensed chromatin to nucleate their formation? What are the functional advantages to having a solid-state genome? We anticipate that the answers to these and many other questions will be forthcoming as studies of the liquid and solid states of chromatin mature over time.

## Data Availability

The datasets in the current study are available from the corresponding author on reasonable request.
